# The efficacy and safety of combined chinese herbal medicine and western medicine therapy for COVID-19: a systematic review and meta-analysis

**DOI:** 10.1186/s13020-022-00600-z

**Published:** 2022-06-21

**Authors:** Lu Li, Hongliang Xie, Ling Wang, Aolin Zhang, Xuan Mou, Yifan Lin, Hongli Ma, Yu Wang, Jian Li, Jingshu Gao, Chi Chiu Wang, Ping Chung Leung, Xiaohui Fan, Xiaoke Wu

**Affiliations:** 1 Pharmaceutical Informatics Institute, College of Pharmaceutical Sciences, Zhejiang University, Hangzhou, 310058 China; 2 Innovation Center in Zhejiang University, State Key Laboratory of Component-Based Chinese Medicine, Hangzhou, 310058 China; 3 Department of Obstetrics and Gynecology, Sir Run Run Shaw Hospital, Zhejiang University School of Medicine, Key Laboratory of Reproductive Dysfunction Management of Zhejiang Province, Hangzhou, 310016 China; 4 Department of Obstetrics and Gynaecology, Li Ka Shing Institute of Health Sciences; School of Biomedical Sciences, Sichuan University-Chinese University of Hong Kong Joint Reproductive Medicine Laboratory, The Chinese University of Hong Kong, Shatin, N.T., Hong Kong China; 5 Institute of Chinese Medicine, The Chinese University of Hong Kong, Shatin, N.T., Hong Kong China; 6 Hangzhou TCM Hospital of Zhejiang Chinese Medical University, Hangzhou, 310053 China; 7 Department of Obstetrics and Gynecology, First Affiliated Hospital, Heilongjiang University of Chinese Medicine, Harbin, 150040 China; 8 Department of Obstetrics and Gynecology, Affiliated Hospital, Guizhou Medical University, Guiyang, 550000 China; 9 College of Basic Medical Sciences, Zhejiang Chinese Medical University, Hangzhou, 310053 China; 10 Heilongjiang Provincial Hospital, Harbin Institute of Technology, Harbin, 150040 China

**Keywords:** Systematic review, Meta-analysis, Combined Chinese Herbal Medicine-Western Medicine therapy, Corona Virus Disease 2019 severity

## Abstract

**Objective:**

To systematically review the clinical efficacy and safety of Chinese herbal medicine (CHM) with and without Western medicine (WM) for different severity of COVID-19.

**Methods:**

CNKI, PubMed, Wanfang Database, ClinicalTrails.gov, Embase, ChiCTR and ICTRP were searched from 01 Jan, 2020 to 30 Jun, 2021. Two authors independently assessed all the randomized clinical trials (RCTs) for trial inclusion, data extraction and quality assessment. Meta-analysis was conducted using Review Manager software (RevMan 5.4.1). Evidence was assessed using Grading of Recommendations Assessment, Development, and Evaluation (GRADE). Primary outcomes included total effectiveness rate. Secondary outcomes included improvements in symptom improvement and total adverse event rate. Different severity of COVID-19 patients was assessed in subgroup analysis. This study was registered with INPLASY, INPLASY202210072.

**Results:**

22 high quality RCTs involving 1789 participants were included. There were no trial used CHM alone nor compare placebo or no treatment. Compared with WM, combined CHM and WM (CHM-WM) treatment showed higher total effectiveness rate, lower symptom scores of fever, cough, fatigue, dry throat and pharyngalgia, shorter mean time to viral conversion, better Computerized Tomography (CT) image and blood results, fewer total adverse events and worse conditions (P < 0.05). Subgroup analysis showed that the total effectiveness rate of combined CHM-WM group was significantly higher than WM group, especially for mild and moderate patients. No significant differences in mortality and adverse events were found between combined CHM-WM and WM treatment. No serious adverse events and long-term outcomes were reported.

**Conclusion:**

Current evidence supported the therapeutic effects and safety of combined CHM-WM treatment on COVID-19, especially for patients with mild and moderate symptoms. Long-term effects of therapy are worthy in further study.

**Supplementary Information:**

The online version contains supplementary material available at 10.1186/s13020-022-00600-z.

## Background

Coronavirus disease 2019 (COVID-19), is a worldwide epidemic with a rapid increase in cases and deaths that posed an enormous threat to public health. COVID-19 was caused by severe acute respiratory syndrome coronavirus-2 (SARS-CoV-2) with high infectivity, which spreads through contact (via larger droplets and aerosols), and longer-range transmission via aerosols, especially in poorly ventilation environment [1, 2]. COVID-19 had a severe influence on people’s health and life, according to the World Health Organization (WHO), as of 4:35 pm CEST on 17 January, 2022, SARS-CoV-2 has infected 326,279,424 individuals worldwide and caused 5,536,609 deaths [3]. Currently, no specific antiviral drugs or efficient vaccines are available to prevent or treat COVID-19 infection, symptomatic and supportive treatments are still the mainstream strategies to manage the infection in clinical practice [4, 5]. Therefore, the effective treatment of COVID-19 is required urgently.

The fundamental pathophysiology of COVID-19 is massive alveolar damage and severe acute respiratory distress syndrome, which are commonly treated by various Western Medicine (WM), including respiratory assisted ventilation, supportive care, anti-infection (mainly antiviral agents) and glucocorticoid therapy, etc. [6–10]. The antiviral agents, such as, alpha interferon (a-INF), remdesivir and arbidol etc. are primarily prescribed [11–14]. However, some antiviral drugs may have potential drug-drug interactions, which may lead to serious adverse drug events or increase the risk of treatment failure [15–17]. In addition, no effective vaccines and specific anti-SARS-CoV-2 agents are available to prevent or treat the disease at present, thus, symptomatic and alternative therapies are urgently needed to manage the infection [18–20]. Chinese Herbal Medicine (CHM), as a complementary and alternative therapy, could inhibit and alleviate excessive immune response and eliminate inflammation via multi-component and multi-target in network pharmacology analysis [21, 22]. CHM exhibited remarkable benefits against the prevention, treatment and rehabilitation of COVID-19 that more than 70,000 infected people have been beneficial from using CHM [23, 24]. A number of CHM formulae and proprietary are recommended for patients with COVID-19 infection by the Chinese Clinical Guidance of COVID-19 Pneumonia Diagnosis and Treatment (Trial Version 8, revised) published by China National Health Commission on April 15, 2021, which include Qingfei Paidu decoction, Xuebijing injection, etc. [25]. Combined CHM-WM may play a pivotal role in alleviating clinical symptoms, decreasing duration of fever and facilitating radiological improvement for COVID-19 [26, 27]. Although several systematic reviews on the efficacy of CHM for the treatment of COVID-19 have been published, their deficiency in methodological have limited their clinical guidance and increased potential bias [28–31]. Besides, there is lack of evidence to support the efficacy of combined CHM-WM for different severity participants. Therefore, comprehensive and rigorous evaluation of clinical research using combined CHM-WM for COVID-19 is needed.

In this study, we aimed to summarize the published high quality randomized clinical trials (RCTs) to evaluate the efficacy of combined CHM-WM therapy for COVID-19 by systematic review and meta-analysis. This study can provide stronger evidence and guidance for the patients, clinicians, researchers and policy makers, which might help to increase better preparation against recurrent outbreaks and inform clinical management across the globe.

## Methods

### Criteria for studies inclusion and exclusion

#### Types of studies

Only RCTs comparing CHM treatment with placebo, no or other treatment for COVID-19 patients were eligible for inclusion.

#### Types of participants

All patients diagnosed with COVID-19 or tested positive were studied, regardless of age, gender, nationality, duration of sickness and severity, etc.

#### Types of interventions

Drug treatments including WM, CHM and other alternative therapies, if possible, either alone or in combination were included. Placebo, no treatment and standard care were included as control.

We excluded the literature if: (1) study types including cohort studies, case reports, case series and revie; (2) acupuncture, psychological supports and other non-pharmaceutical treatment were performed; (3) duplicate publications; (4) non-COVID-19 participants were enrolled.

#### Types of outcome measures

All efficacy and safety relevant outcomes reported in the included RCTs were checked and summarized. Primary outcomes included the total effectiveness rate. Secondary outcomes included the effectiveness relevant index such as symptom improvement, virological outcome, Computerized Tomography (CT) image improvement rate, blood test improvement, and safety relevant index such as total adverse event rate, adverse event rate, worse condition rate and mortality.

$${\text{Total effectiveness rate }} = \, \left( {{\text{clinical recovery cases }} + {\text{ significantly effective cases }} + {\text{ effective cases}}} \right) \, /{\text{ total cases }} \times { 1}00\% ;$$

Symptom improvement refers to the CHM symptom score of different clinical symptoms which was based on the Guiding Principles for Clinical Research of New Chinese Medicine (the 2010 revision) [32]. In which, when the data was reported as ‘media, IQR’, it was converted into ‘mean ± SD’ through mathematical methods [33, 34] and included it in the meta-analysis; Virological outcome refers to the time of novel coronavirus nucleic acid changes from positive to negative after the treatment;

Chest CT images improvement was defined as a decreased area of any radiologic abnormality, infiltration or decreased density of the ground-glass opacity or nodules;

Blood test improvement refers to the proportion of patients whose blood sample index, such as, white blood cell count (WBC), lymphocyte absolute value (LYM), lymphocyte ratio (LYM%), c-reactive protein (CRP) and procalcitonin (PCT) etc. returns to normal after treatment accounts for the total number of patients;

$${\text{Total adverse event rate }} = {\text{ adverse event cases }} + {\text{ serious adverse event cases }}\left( {{\text{SAE}}} \right) \, + {\text{ worse condition }}\left( {{\text{convert to severe cases and }}/{\text{ or critical illness cases}}} \right) \, + {\text{ mortality cases}}) \, /{\text{ total cases }} \times { 1}00\% ;$$

$${\text{Adverse event rate }} = {\text{ adverse event cases }}/{\text{ total cases }} \times { 1}00\% ;$$

$${\text{Worse condition rate }} = {\text{ convert to severe cases and }}/{\text{ or critical illness cases }}/{\text{ total cases }} \times { 1}00\% ;$$

$${\text{Mortality rate }} = {\text{ mortality cases }}/{\text{ total cases }} \times { 1}00\% .$$

### Literature search

Databases included the China National Knowledge Infrastructure (CNKI), PubMed, Wanfang Database, ClinicalTrails.gov, Chinese Clinical Trial Registry (ChiCTR), Embase and International Clinical Trials Registry Platform (ICTRP) were searched from 01 Jan, 2020 to 30 Jun, 2021 for all published RCTs. Search strategies were designed with terms related to COVID-19, CHM, WM, etc. We prospectively submitted the systematic review protocol for registration on INPLASY (INPLASY202210072). This review was structured in accordance with the PRISMA checklist 2020. (See Additional files: Additional files 1, Additional file 2, Additional file 3, Additional file 4). There was no limitation on language of the publications.

### Selection of studies

**Fig. 1:**
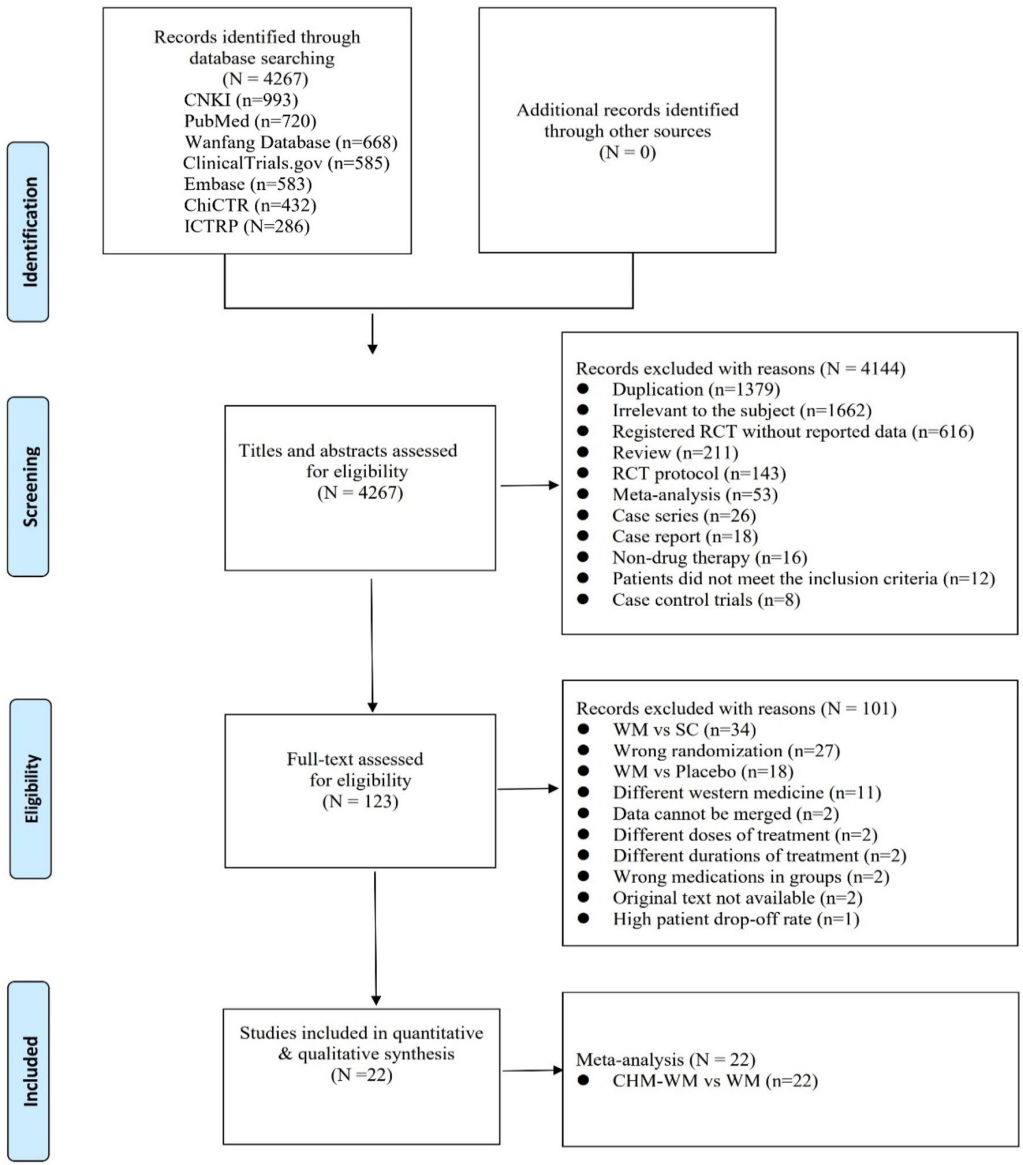
Flow diagram of study selection

Search results were screened and confirmed by two authors independently. Any disagreements were resolved through discussion or consultation with the third assessor. A study flow diagram was created to map out the number of records identified, included and excluded (Fig. 1).

### Data collection and extraction

We designed a form to extract data, including baseline characteristic of the participants, study design, intervention and comparator characteristic and relevant clinical outcomes. For eligible studies, two review authors completed the agreed data extraction form independently. We resolved discrepancies through discussions or consulted a third person. We entered data into Review Manager software (RevMan 5.4.1) and checked for accuracy. When information regarding any of the above is unclear, we attempted to contact authors of the original reports to provide or confirm further details. Outcome data were extracted for further meta-analysis.

### Risk of bias assessment

Two review authors independently assessed risk of bias for each study using the criteria outlined in the Cochrane Handbook for Systematic Reviews of Interventions (Higgins 2011) [35]. It included: (1) random sequence generation (checking for possible selection bias); (2) allocation concealment (checking for possible selection bias); (3) blinding of participants and personnel (checking for possible performance bias); (4) blinding of outcome assessment (checking for possible detection bias); (5) incomplete outcome data (checking for possible attrition bias due to the amount, nature and handling of incomplete outcome data) and (6) selective reporting (checking for reporting bias); (7) publication status (checking for publication bias). We resolved any disagreement by discussion or by involving a third assessor.

### Data analysis and synthesis

#### Measures of treatment effect

We used Review Manager software (RevMan 5.4.1, 2020) for statistical analysis. For dichotomous data, we presented results as relative risk (RR) ratio with 95% confidence intervals (CIs). For continuous data, we used the mean difference (MD) if outcomes were measured in the same way between trials. We calculated the standardized mean difference (SMD) to combine trials that measure the same outcome, but use different methods. In cases where trial data were missing, we first attempted to contact the original trial investigator to verify the study characteristics and obtain missing information. If the missing data are not available, then we would base on the number randomized minus any participants with missing outcomes. We excluded trials where more than 20% of participants were lost to follow-up. χ^2^ and I^2^ quantitative tests were used to test the heterogeneity among the studies. When P < 0.10, I^2^ > 50%, a random-effects model was selected for meta-analysis, and when P > 0.10, I^2^ < 50%, a fixed-effect model was applied. Sensitivity analyses were performed by excluding a study and analyzing the remaining data for each round to test the robustness of our results. Reporting bias (such as publication biases) was reported by using funnel plots in the meta-analysis when the number of trials on an outcome measure was larger than ten.

#### Subgroup analysis

Subgroup analyses including total effectiveness rate and total adverse event rate of different severity of COVID-19 patients between groups were recorded. We would report the results of subgroup analyses quoting the Chi^2^ statistic and P value, and the interaction test I^2^ value. The classification of different cases was shown in Table 1.

**Table 1:** Classification of different cases of COVID-19

Type of participants		Clinical symptoms
Severe cases	Adult case	(i) Respiratory distress (≧30 breaths/ min)
		(ii) Oxygen saturation ≤ 93% at rest
		(iii) Arterial partial pressure of oxygen (PaO2)/ fraction of inspired oxygen (FiO2)≦ 300 mmHg (l mmHg = 0.133 kPa)
		(iv) Chest imaging shows obvious lesion progression within 24–48 h > 50%
	Child cases	(i) High fever lasting more than three days
		(ii) Tachypnea, independent of fever and crying
		(iii) Oxygen saturation ≤ 93% on finger pulse oximeter taken at rest
		(iv) Labored breathing
		(v) Lethargy and convulsion
		(vi) Difficulty feeding and signs of dehydration
Moderate cases		(i) Fever
		(ii) Respiratory symptoms
		(iii) Radiological findings of pneumonia
Mild cases		(i) the mild symptom improvement
		(ii) No sign of pneumonia on imaging

### Quality of evidence assessment

Two review authors, who were not involved in all included studies, assessed the quality of evidence using the Grades of Recommendations Assessment, Development and Evaluation (GRADE) approach suggested by GRADE Working Group in order to assess the quality of the body of evidence [36, 37]. Where data are available, GRADE was used to assess the overall quality of the evidence for WM intervention alone versus combined CHM-WM intervention.

We used the GRADEpro Guideline Development Tool to import data from RevMan 5.4 in order to create’Summary of findings’ tables [38]. A summary of the intervention effect and a measure of quality for WM treatment alone versus combined CHM-WM treatment were produced using the GRADE working group’s approach. The GRADE approach uses five considerations (study limitations, consistency of effect, imprecision, indirectness and publication bias) to assess the quality of the body of evidence for each outcome. The evidence can be downgraded from ‘high quality’ by one level for serious (or by two levels for very serious) limitations, depending on assessments for risk of bias, indirectness of evidence, serious inconsistency, imprecision of effect estimates or potential publication bias [36, 37].

## Results

### Literature screening

A total of 4267 clinical studies from different databases were identified by literature search. 4144 trials were excluded initially according to the inclusion and exclusion criteria after screening the titles and abstracts. Full texts of 123 studies were further reviewed, and 101 studies were further excluded with reasons as follows: 34 studies compared WM with standard care (SC); 27 studies had wrong randomization; 18 studies compared WM with placebo; 11 studies used different Western medicine between two groups, 4 studies showed different doses and durations of WM treatment; 2 studies had wrong medications in groups; 2 studies without original text and 1 study had high patient drop-off rate. In total, 22 RCTs involving 1789 participants comparing combined CHM-WM with WM were finally included for meta-analysis [39–60]. There were no trial used CHM alone nor compare placebo or no treatment. Figure 1 summarized the process for the study selection. A summary of the characteristics of 22 RCTs involving 1789 participants comparing combined CHM-WM with WM is shown in Table 2.

**Table 2:** Summary of study characteristics for included studies

Author	Randomization	COVID-19 type	Age (M ± SD)	Sex		Disease course	Sample size (T/C)	Intervention (dose, dosing & duration)		Outcomes			
				Male	Female			Treatment (T)	Control (C)	Efficacy	P value	Safety	P value
Chen. 2021 [39]	Random number table method	Mild/ Moderate	T: 19–61(50.16 ± 5.11) C: 20–62(49.52 ± 5.06)	T: 17 C:18	T:13 C:12	NR	30/30	Lianhua Qingwen Capsules 4 capsules/time, oral, tid, medication until discharge plus control	Recombinant human interferon α2b (5 million U, arm, bid, < 10d); Lopinavir and tonavir tablets (2 slices/time, oral, bid, < 10d)	①↓: Turn severe rate	P < 0.05	Nausea and vomiting	P > 0.05
										②↓: Time for group fatigue, cough, shortness of breath and other symptoms to disappear	P < 0.05	Mild diarrhea	P > 0.05
										③Clinical recovery time, nucleic acid turning negative time	P > 0.05	Liver damage	P > 0.05
										④Time for fever to subside	P > 0.05	Dizziness	P > 0.05
										⑤↓: IL-10, PCT, hs-CRP	P > 0.05		
Chen. 2020 [40]	Random number table method	Confirmed	T: 26–58(42.6 ± 3.5) C: 24–59(43.1 ± 3.2)	T:8 C:9	T:7 C:6	T: 4-18d(6.9 ± 1.5)d C: 3-19d(6.2 ± 1.4)d	15/15	Xuebijing Injection 10 ml, oral, bid, 14d plus control	Ifn-α, lopinavir, ritonavir	①↑: Total effectiveness rate	P < 0.05	NR	NR
										②Complications of patients	P > 0.05		
										③Serum CRP levels before treatment	P > 0.05		
										④↓: Serum CRP levels after treatment	P < 0.05		
Duan. 2020 [41]	SPSS random number table grouping (2: 1)	Mild	T: 51.99 ± 13.88 C: 50.29 ± 13.17	T: 39 C: 23	T: 43 C: 18	NR	82/41	Jinhua Qinggan Granules 10 g, oral, tid, 5d plus control	Antivirals or antibiotics	①↓: Disappearance rate of clinical symptoms	P > 0.05	↑: Diarrhea	P < 0.01
										②↓: TCM symptoms score	P < 0.05		
										③Hospitalization rate	P > 0.05		
										④Hamilton anxiety scale score	P < 0.01		
Fu 2020a [42]	Random number table method	Mild/ Moderate	T: 29–65(43.26 ± 7.15) C: 30–70(43.68 ± 6.45)	T: 17 C: 19	T: 15 C: 14	T:7.56 ± 1.25 C: 8.47 ± 1.35	32/33	Toujie Quwen Granules no, oral, bid, 10d plus control	Abidol (0.2 g, oral, tid, 10d), Moxifloxacin (0.4 g, oral, qid, 10d); Ambroxol (0.03 g, oral, tid, 10d)	①↓: Curative effect of patients after treatment	P = 0.022	NR	NR
										②↓: Scores of TCM syndrome in two groups after treatment	P < 0.05		
										③Curative effect of chest CT in two groups after treatment	P > 0.05		
										④WBC, LYM% after treatment	P < 0.05		
										⑤↓: CRP, PCT in two groups after treatment	P < 0.05		
										⑥D-Dimer in two groups after treatment	P > 0.05		
										⑦↑: LYM cell counts, ↓: NEU% in two groups after treatment	P < 0.05		
Fu 2020b [43]	NR	Moderate	T: 29–65(45.26 ± 7.25) C: 30–70(44.68 ± 7.45)	T: 19 C: 19	C: 18 C: 17	T: 7.56 ± 1.25 C: 8.47 ± 1.35	37/36	Toujie Quwen Granules no, oral, bid, 15d plus control	Abidol (0.2 g, oral, tid, 10d); Ambroxol (0.03 g, oral, tid, 15d)	①↓: The effect of treatments and discharge rate	P < 0.05	NR	NR
										②↓:The scores of TCM syndrome	P < 0.05		
										③↑: LYM cell counts	P < 0.05		
										④↓: Changes of CRP	P < 0.05		
										⑤WBC, LYM%	P > 0.05		
He 2021 [44]	Random number table method	Mild	NR	NR	NR	NR	36/36	Buzhong Yiqi Decoction 1 dose, oral, bid, 10d plus control	Abidor(0.2 g, oral, tid, 5d)	①↓:The scores of TCM syndrome	P < 0.05	NR	NR
										②↑: Curative effect	P < 0.05		
										③↓: hs-CRP、ESR	P < 0.05		
										④↓: IL-6	P > 0.05		
										⑤Blood analysis, PCT	P > 0.05		
Hu 2021 [45]	Block random method (1:1)	Confirmed	T: 50.4 ± 15.2 C: 51.8 ± 14.8	T: 79 C: 63	T: 71 C: 71	T: 9.5 ± 5.1 C: 9.9 ± 5.9	142/142	Lianhua Qingwen Capsules 4 capsules, oral, tid, 14d plus control	Oxygen therapy, antiviral medications, systemic corticosteroids	①↑: Rate of symptoms recovery at day 14	P = 0.022	Abnormal liver function	P = 1.000
										②↓: Median Time to symptom recovery	P < 0.001	Renal dysfunction	P = 0.476
										③↑: Rate of recovery of chest CT manifestations	P < 0.001	Headache	P = 1.000
										④↑: Overall rate of clinical cure	P < 0.05	Nausea	P = 0.758
										⑤Conversion rate of SARS-CoV-2 viral assay findings	P = 0.279	Vomiting	P = 0.652
										⑥Median viral assay conversion time	P = 0.151	Diarrhea	P = 0.026
												Loss of appetite	P = 0.584
Lin 2020 [46]	Random number	Moderate	T: 20–80(46.02 ± 12.09) C: 26–67(43.08 ± 12.34)	T: 15 C: 23	T: 26 C: 18	T: 1–14(5.07 ± 3.467) C: 1–17(6.02 ± 3.698)	41/41	Xuanfei Qingre Decoction 0.4 g, oral, qd, 5d plus control	Ifn-α (5 million U, aerosol inhalation, bid, 10d); Lopinavir/Litonavir (0.4 g/0.1 g oral, bid,14d), bed rest, support treatment	①↓: Cough score and total score of TCM syndromes	P < 0.05	Turn to severe group (T:0; C:2); critical group (T:0, C:0)	P > 0.05
										②↓: Days for sputum nucleic acid turns cloudy, hospitalization days; hospitalization expenses	P < 0.05		
										③↓: Days for symptoms to disappear	P < 0.05		
										④↑: Absorption effect of lung lesions	P = 0.003		
										⑤↑: TCM symptom curative effect	P = 0.002		
										⑥Fever, fatigue, sore throat, chest tightness, anorexia, diarrhea	P > 0.05		
										⑦Remaining indicators	P > 0.05		
										⑧↑: CRP, ALT, AST, ESR	P < 0.05		
Liu 2021 [47]	Random number table method	Mild	T: 15–80(48.51 ± 4.56) C: 18–79(48.43 ± 4.52)	T: 16 C: 15	T: 28 C: 29	NR	44/44	Lianhua Qingwen Capsule 1.4 g, oral, tid, 21d Pneumonia Agreement 2 1 dose, oral, bid, 21d plus control	Abidor(0.2 g, oral, tid, 21d); Oseltamivir(0.015 g, oral, bid, 21d)	①↑: Total effectiveness rate for clinical efficacy	P = 0.011	↓: Diarrhea	NR
												↓: Headache	NR
												↓: Dizziness	NR
												↓: Nausea and vomiting	NR
												↓: Total adverse rate	P = 0.02
Liu 2021 [48]	NR	Severe	T: 26–70(48.0 ± 1.6) C: 27–70(48.5 ± 1.3)	T: 14 C: 13	T: 11 C: 12	NR	25/25	Huashi Baidu Recipe 1 ~ 2 dose/day, oral,100 ~ 200 m/time, oral or nasal feeding, bid ~ qid, 21d plus control	Tocilizumab (0.004 ~ 0.008 g/kg for the first dose, 0.4 g for the recommended dose, iv drop, bid, 30d)	①↑: Clinical efficacy	P < 0.05	↓: Gastrointesti-nal hemorrhage	P < 0.05
										②↑: WBC, LYM	P < 0.05	↓: Clotting time	P < 0.05
										③↓: CRP, ESR	P < 0.05	↓: Total adverse event rate	P < 0.05
Wang 2020 [49]	Random allocation, (1:1)	Moderate	T: 21–65(43.75 ± 4.10) C: 23–63(42.56 ± 4.58)	T: 23 C: 25	T: 22 C: 20	T: 2–14(7.50 ± 2.42) C: 2–15(7.18 ± 2.16)	45/45	Bifidobacterium Tablets 3 tablets, oral, tid, 15d Sanren decoction 1 pay, oral, tid,15d plus control	Oseltamivir capsule (0.1 g, oral, tid, 15d)	①↓:Clinical symptom score	P < 0.05	NR	NR
										②↓: Severe transfer rate	P > 0.05		
										③↑: Total effectiveness rate	P > 0.05		
										④↓: blood CRP, PCT, D-dimer level	P < 0.05		
Wang 2021 [50]	NR	Moderate	T: 18–80(48 ± 13.2) C: 19–79(49.4 ± 13.3)	T: 35 C: 36	T: 35 C: 34	NR	70/70	Qingfei Paidu Decoction 100 mL, oral, bid, 10d plus control	Moxifloxacin hydrochloride tablets (0.4 g, oral, qd, 10d); Arbidol hydrochloride dispersible tablets (0.2 g, oral, tid, 10d)	①↑: Clinical efficacy	P < 0.05	Palpitations (T:C = 0:2)	NR
										②↓: TCM syndrome score	P < 0.05	Nausea (T:C = 1:4)	NR
										③↓: Hospitalization days	P < 0.05	Fatigue (T:C = 1:3)	NR
										④↑: Inflammation absorption	P < 0.05	↓: total adverse event rate	P < 0.05
										⑤↓: WBC, LYM%, CRP	P < 0.05		
Wang 2020a [51]	Random allocation, (1:1:1)	Confirmed	T1: 35–70(39.24 ± 10.01) T2:43–71(54.90 ± 3.61) C: 29–66(55.90 ± 3.71)	T1: 5 T2:4 C: 5	T1: 5 T2: 6 C: 5	NR	10/10/10	Traditional Chinese Medicine no, oral + fume absorption, bid, 7d plus control TCM + Vitamin C no, oral + fume absorption, bid, 7d plus control	Ribavirin, anti-infection, auxiliary support drugs	①Time of improvement of fatigue, cough, dry throat, and shortness of breath	P < 0.05 (A-C)	NR	NR
										②Therapeutic effects on symptoms	P = 0.014 (A-B) P = 0 (A-C) P = 0.11 (B-C)		
										③Nucleic acid turns negative	P > 0.05		
										④Time of improvement of fatigue, cough, dry throat, and shortness of breath	P > 0.05		
Wang 2020b [52]	Random number table method	Moderate	T: 4–70(43.43 ± 17.51) C: 6–67(41.73 ± 15.16)	T: 6 C: 5	T: 5 C: 6	T: 1–21(6.5 ± 4.3) C: 1-18d(41。73 ± 15.16)	11/11	Qingre Kang oral liquid 20 ml, oral, tid, 10d plus control	Recombinant human interferon α2b injection (5 million U, injection, bid, 10d); Arbidol hydrochloride (0.2 g, oral, tid,10d)	①↑: Fever, cough and fatigue disappearance rate, CT findings	P > 0.05	NR	NR
										②↓: Duration of fever	P > 0.05		
										③↑: Lung CT lesion absorption	P > 0.05		
Wen 2020 [53]	Random number table method	Severe	T: 49.1 ± 4.8(50 ml);47.1 ± 5.2(10 ml) C: 47.7 ± 5.7	T:11(50 ml) T:12(50 ml) C: 9	T:8(50 ml) T:9(50 ml) C:11	NR	20/20/20	Xuebijing Injection 50 ml, oral, bid, 7d plus control Xuebijing Injection 100 ml, oral, bid, 7d plus control	Did not state details	①↑: Condition improvement	P < 0.05	NR	NR
										②Nucleic acid turns negative	P > 0.05		
										③↓: CRP, ESR	P < 0.05		
										④↓: APACHEIIscore	P < 0.05		
										⑤↑: WBC	P < 0.05		
Ye 2020 [54]	Random allocation (1:2)	Severe	T: 53–69 C:47–67	T: 2 C: 4	T: 25 C: 10	T: 9(6.5–11.5) C: 9.5(6–14)	28/14	Maxinshigantang–Dayuanyin Decoction or Shengfutang Decoction 200 ml, oral, bid, 7d plus control	Antivirals, antibiotics, immune modulators, systemic corticosteroids	①Clinical improvement	P = 0.350	Mortality rate	P = 0.454
Yu 2020 [55]	Random number table method	Mild/ Moderate	T: 48.27 ± 9.56 C: 47.25 ± 8.67	T: 82 C: 89	T: 65 C: 59	NR	147/148	Lianhua Qingwen Granules 6 g, oral, tid, 7d plus control	Abidol hydrochloride dispersible tablets (0.2 g, oral, tid, 14d), Moxifloxacin hydrochloride tablets (0.4 g, oral, qd,14d), Ambroxol hydrochloride (0.03 g, oral, tid,14d)	①↑: Clinical efficacy	P < 0.05	NR	NR
										②↓: Severe disease rate	P < 0.05		
										③↓: TCM syndrome score	P < 0.05		
										④Chest CT effectiveness	P > 0.05		
										⑤↑: WBC, LYM	P > 0.05		
										⑥↓: CRP, PCT	P < 0.05		
Zhang 2020 [56]	NR	Moderate	T: 53.7 ± 3.5 C: 55.6 ± 4.2	T: 9 C: 13	T: 10 C: 13	NR	22/23	Modified Dayuan formula no, oral, tid, 7d plus control	Oxygen therapy, symptomatic treatment, antivirus treatment	①↑: Clinical efficacy	P < 0.05	NR	NR
										②↑: Chest CT improvement rate	P < 0.05		
										③↑: LYM%	P < 0.05		
										④↓: CRP	P < 0.05		
										⑤WBC	P > 0.05		
Zhang 2020 [57]	Random allocation, (1:2)	Moderate	T: 53.4 ± 13.70 C: 52.0 ± 14.10	T: 50 C: 23	T: 30 C: 17	T: 2.65 ± 1.45 C: 2.41 ± 1.59	80/40	Jinyinhua oral liquid 60 ml, oral, tid, 10d plus control	Lopinavir (0.4 g, oral, bid, 10d); Ritonavir (0.1 g, oral, bid, 10d); Ifn-α (5 million U, injection, bid, 10d)	①↑: Disappearance rate of clinical symptoms	P < 0.05	Mild diarrhea (1:0)	P > 0.05
										②↓: Chest CT examination score after treatment for 10d	P < 0.01	Total adverse event rate	P > 0.05
										③Negative conversion rate of SARS-CoV-2-qRT-PCR	P > 0.05		
Zhao 2020 [58]	Random selection	Severe	NR	T: 8 C: 14	T: 7 C: 10	T:6.5(4.0–7.5) C:5.5(3.0–7.0)	15/24	Yidu-toxicity blocking lung Decoction no, oral, no,14d plus control	Did not state details	①Cure rate	P = 0.878	NR	NR
										②Hospital stay	P = 0.662		
										③↑: TNF-α	P = 0.035		
										④↑: IL-6	P = 0.013		
										⑤Spearman analysis for CD4 and IL-6	P = 0.772		
Zheng 2020 [59]	Random number table method	Confirmed	T: 52.47 ± 9.87 C: 52.47 ± 10.99	T: 32 C: 20	T: 28 C: 24	NR	40/40	Sodium tanshinone IIA sulfonate 0.06 g, oral, qd, 10d plus control	Oxygen inhalation, fluid rehydration, anti-inflammatory, antivirals, ventilator assisted breathing, psychological guidance, vital signs monitoring	①↑: Total effectiveness rate	P < 0.05	NR	NR
										②↑: Level of lung function	P < 0.05		
										③↓: Levels of inflammatory factors	P < 0.05		
Zhou 2020 [60]	Random number table method	Moderate	T: 25–63(42.17) C: 12–77(40.88)	T: 8 C: 9	T: 9 C: 8	NR	52/52	Diammonium Glycyrrhizinate enteric coated capsules 0.15 g, oral, qid, 14d plus control	Lopinavir tablets / ritonavir tablets (0.5 g, oral, bid,14d)	①↑: Cure rate, excellent, total effectiveness rate	P < 0.05	Nausea and vomiting (T:C = 4:3)	NR
										②↓: CRP, IL-4, TNF-α	P < 0.05	Diarrhea (T:C = 3:3)	NR
										③↑: CD3^+^ CD4^+^, CD8^+,^ CD4^+^/CD8^+^	P < 0.05	Abnormal liver function (T:C = 1:9)	NR
												↓: Total adverse event rate	P = 0.013

↓ decreased; ↑: increased, *Qd* Once a day, *Bid* Twice a day, *Tid* three times a day, *Qid* four times a day, *NR* not reported, *TCM* Traditional Chinese medicine, *WBC* White blood cell count, *LYM* Lymphocyte absolute value, *LYM%* Lymphocyte ratio, *CRP* c-reactive protein, *PCT* Procalcitonin, *RT-PCR* Reverse transcription-polymerase chain reaction, *IL-10* Interleukin-10, *IL-6* Interleukin-6, *IL-4* Interleukin-4, *hs-CRP* Hypersensitive-c-reactive-protein, *NEU* Neutrophils, *ALT* Alaninetransaminase, *AST* Glutamic oxaloacetic transaminase, *ESR* Erythrocyte sedimentation rate, *APACHE-II* Acute physiology and chronic health evaluation, *CD* Cluster of differentiation, *TNF-α* Tumor necrosis factor

### Results on efficacy (combined CHM-WM vs WM)

#### Total effectiveness rate (I^2^ < 30%, P < 0.05)

**Fig. 2:**
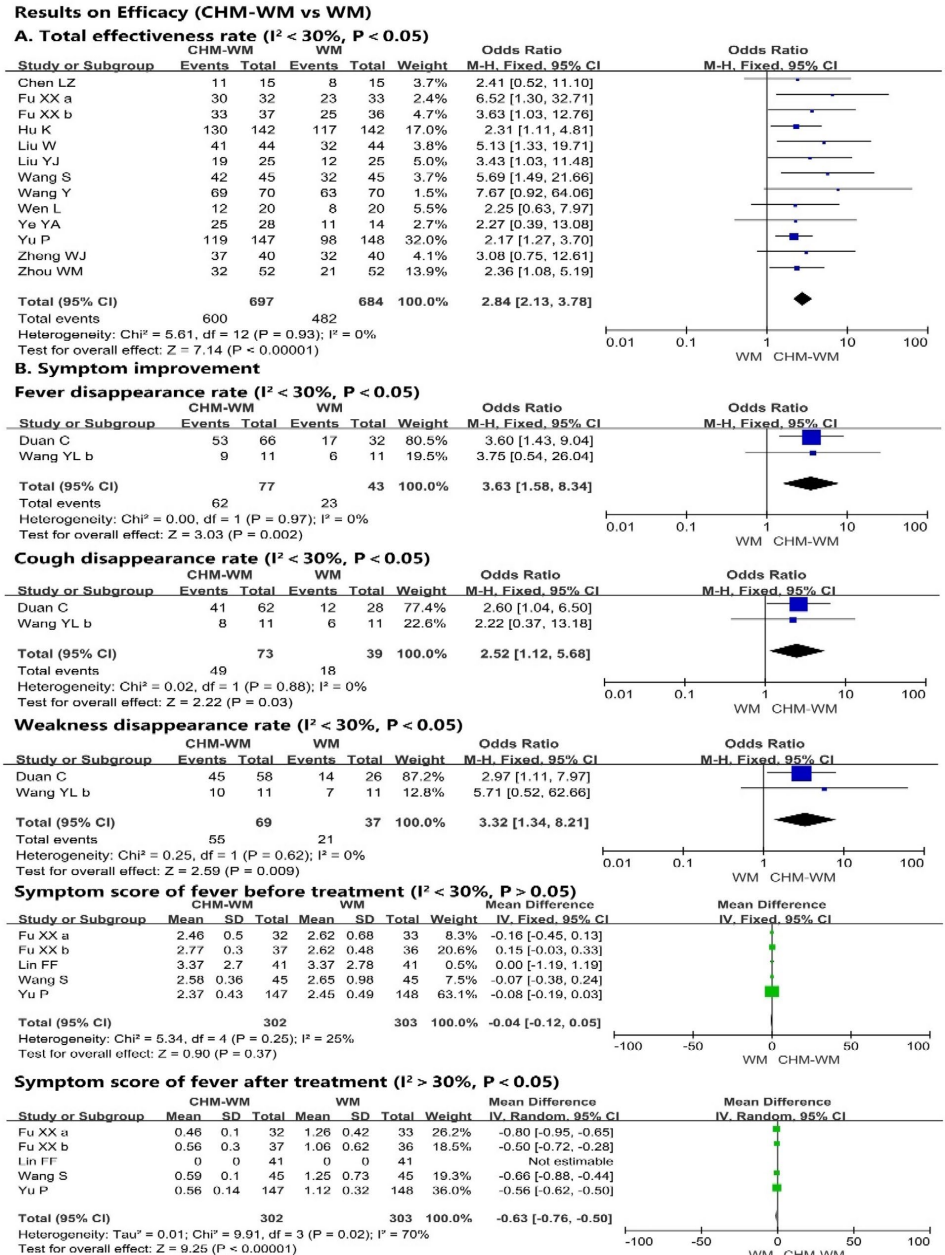

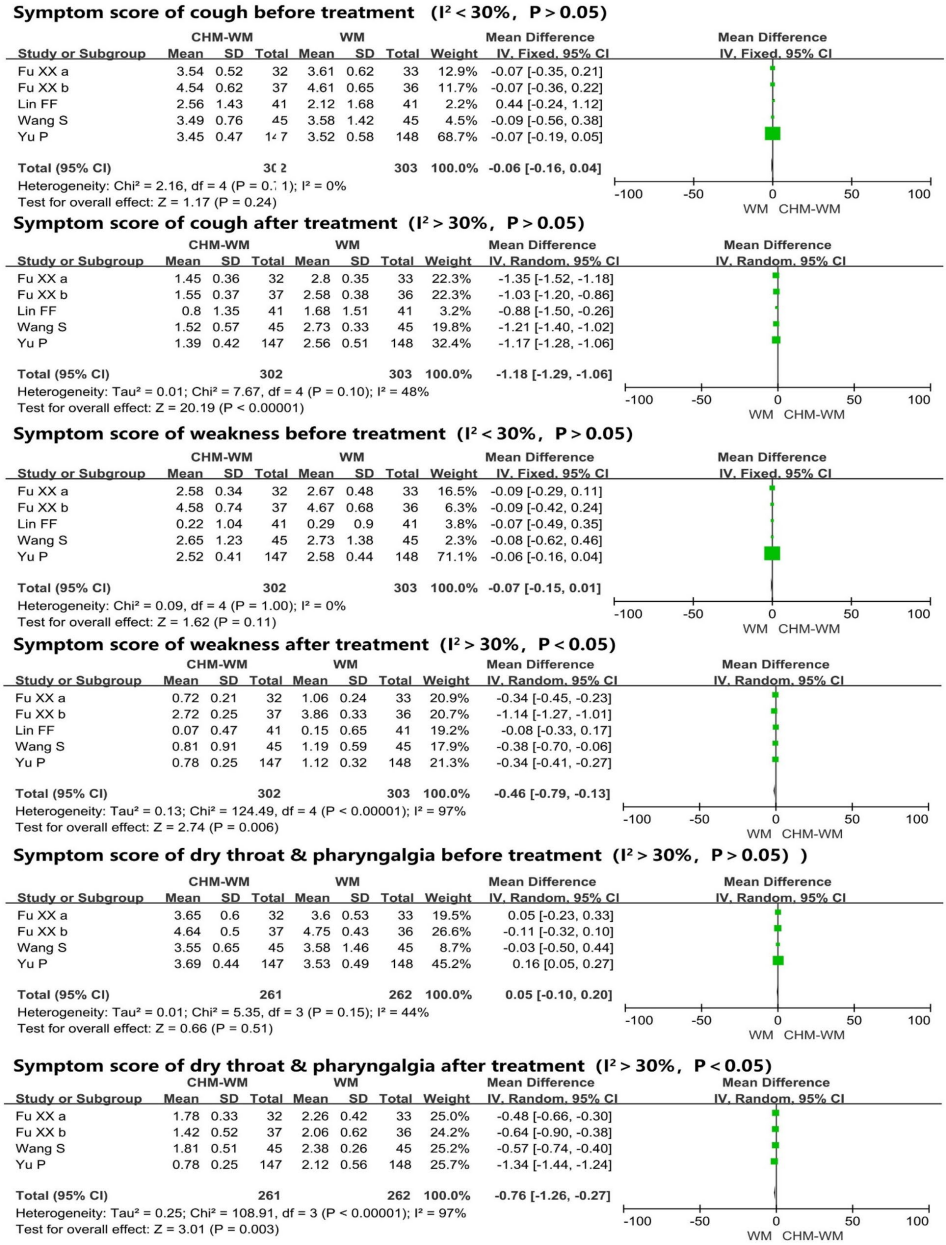

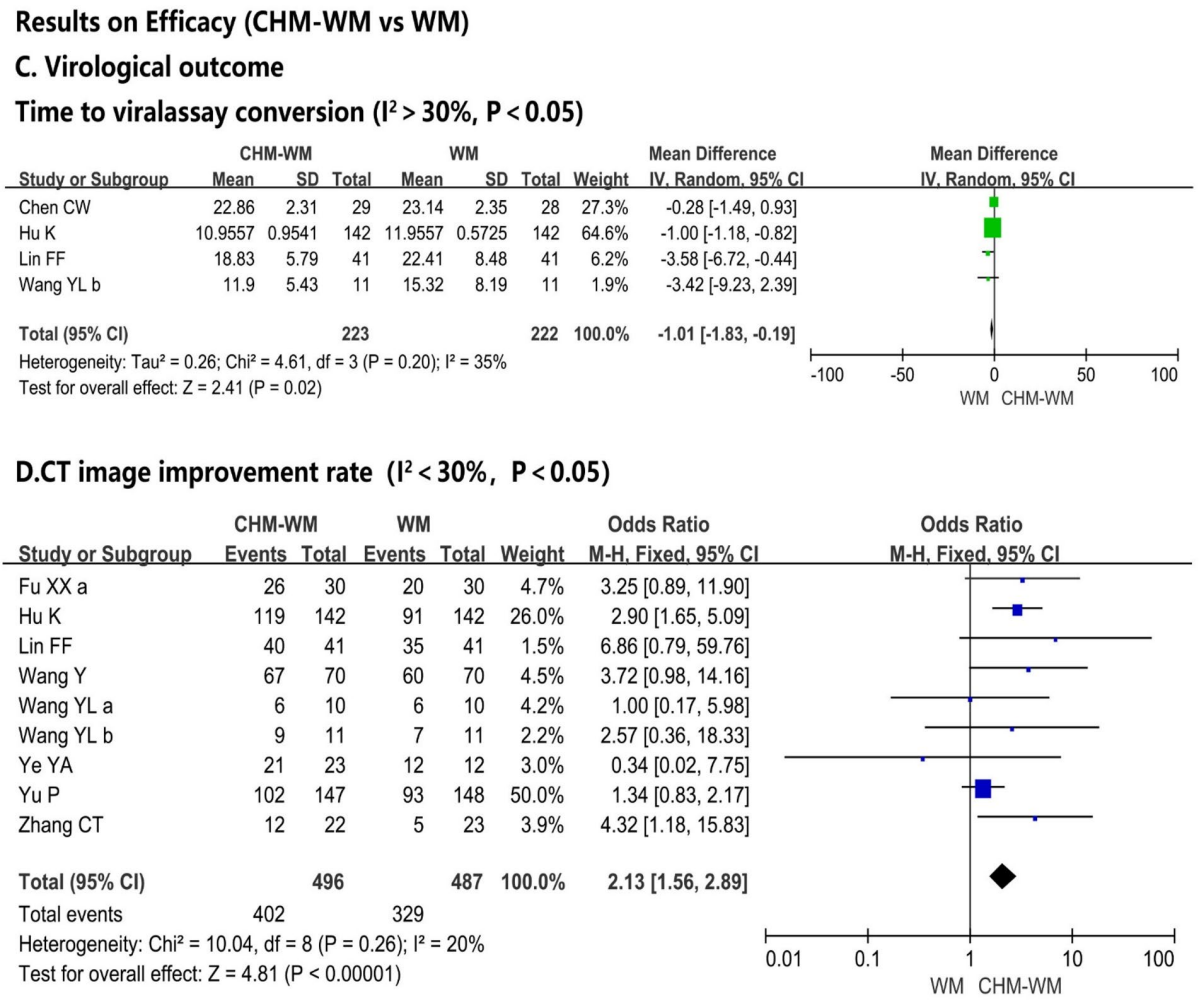

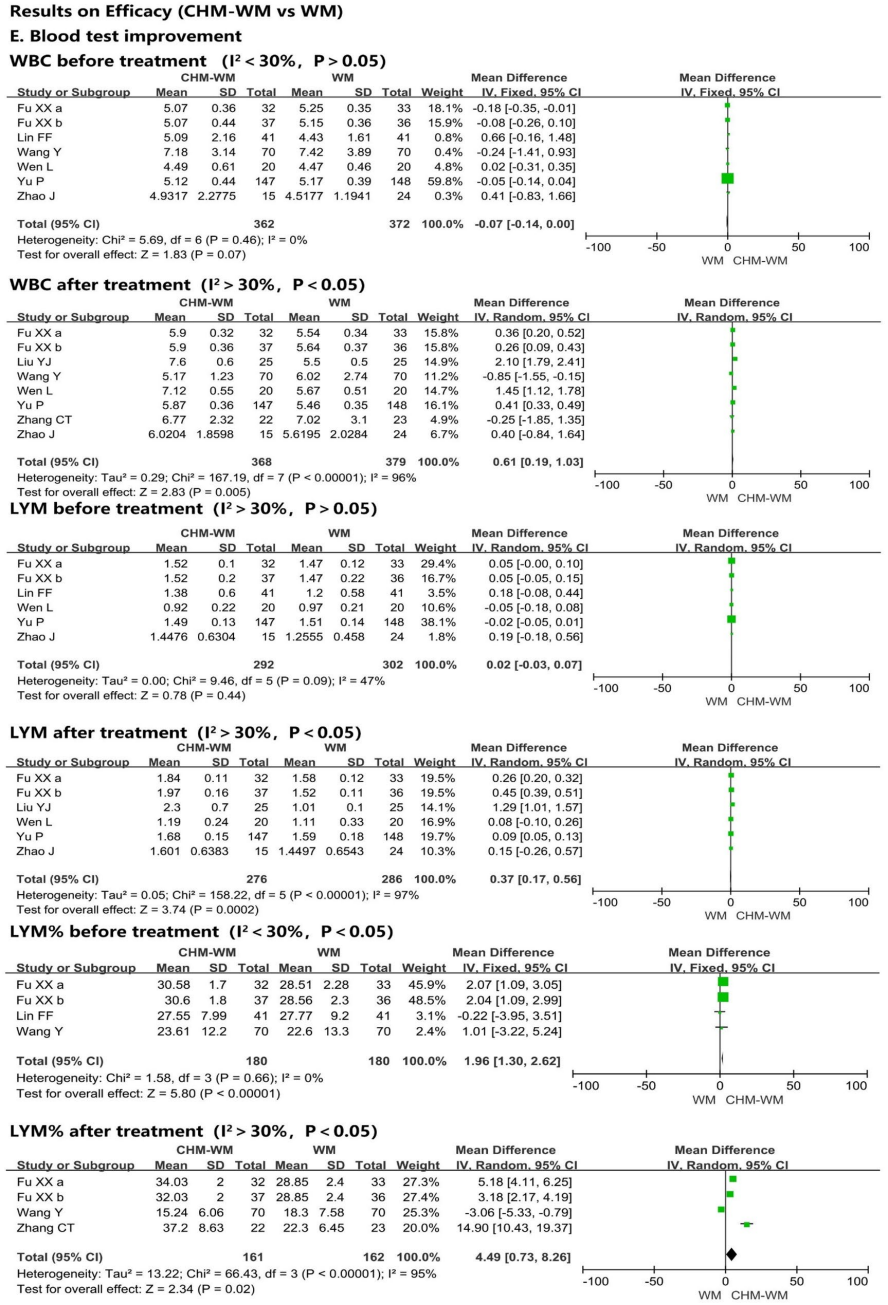

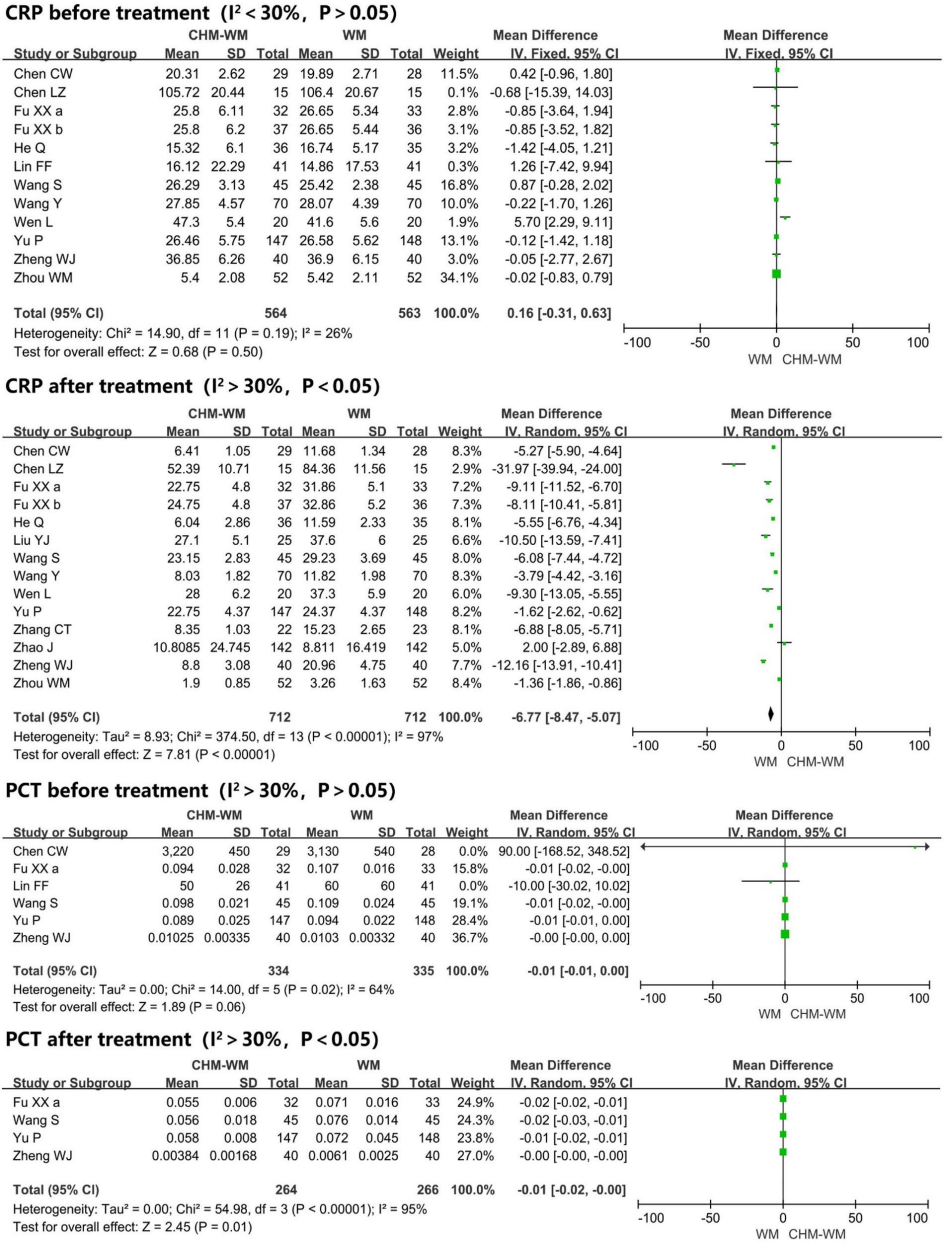
**A**, **B**. Forest plot of the efficacy of combined CHM-WM vs WM. **C**, **D **Forest plot of the efficacy of combined CHM-WM vs WM. **E **Forest plot of the efficacy of combined CHM-WM vs WM

Thirteen trials [40, 42, 43, 45, 47–50, 53, 55, 59, 60] reported that the total effectiveness rate after treatment was significantly increased in combined CHM-WM group compared with WM group (P < 0.00001, Odds ratio (OR) = 2.84, 95% confidence ratio (CI) = 2.13 – 3.78, Fig. 2A).

#### Symptom improvement

Six trials [41, 43, 46, 49, 52, 55] reported the clinical symptoms (Fig. 2B). Two of these trials [41, 52] showed the fever, cough and weakness disappearance rate were improved significantly in combined CHM-WM group compared with WM group (P = 0.002, OR = 3.63, 95% CI = 1.58–8.34; P = 0.03, OR = 2.52, 95% CI = 1.12–5.68; P = 0.009, OR = 3.32, 95% CI = 1.34–8.21). In addition, five trials [42, 43, 46, 49, 55] reported no significant differences in fever, cough and weakness between two groups before treatment (P = 0.37, Mean difference (MD) = − 0.04, 95% CI = − 0.12 to 0.05; P = 0.24, MD = − 0.06, 95% CI = − 0.16 to 0.04; P = 0.11, MD = − 0.07, 95% CI = − 0.15 to 0.01). However, from the forest plot of five trials [42, 43, 46, 49, 55], the fever, cough and weakness symptoms after treatment were significantly lower in combined CHM-WM group compared with WM group (P < 0.00001, MD = − 0.63, 95% CI = − 0.76 to -0.50; P < 0.00001, MD = − 1.18, 95% CI = − 1.29 to − 1.06; P = 0.006, MD = -0.46, 95% CI = − 0.79 to − 0.13). Meanwhile, four trials [42, 43, 49, 55] reported no significant difference in the dry throat & pharyngalgia symptom between two groups before treatment. However, after treatment, the symptoms decreased significantly in combined CHM-WM group compared with WM group from the forest plot of four trials [42, 43, 49, 55] (P = 0.51, MD = 0.05, 95% CI = − 0.10 to 0.20; P = 0.003, MD = − 0.76, 95% CI = − 1.26 to − 0.27).

#### Virological outcomes (I^2^ > 30%, P < 0.05)

Four trials [36, 45, 46, 52] evaluated the virological outcomes (Fig. 2C). The mean time to viral assay conversion was increased significantly in WM group compared with combined CHM-WM group (P = 0.02, MD = − 1.01, 95% CI = − 1.83 to − 0.19).

#### CT image improvement rate(I^2^ < 30%, P < 0.05)

The improvement rate of CT after the intervention was significant higher in combined CHM-WM group compared with the WM group (P < 0.0001, OR = 2.13, 95% CI = 1.56–2.89, Fig. 2D) according to the nine trials [42, 45, 46, 50–52, 54–56].

#### Blood test improvement

Fifteen trials [36, 40, 42–44, 46, 48–50, 53, 55, 56, 58–60] reported the blood test improvement (Fig. 2E). Of these, seven trials [42, 43, 46, 50, 53, 55, 58], six trials [42, 43, 46, 53, 55, 58], and twelve trials [36, 40, 42–44, 46, 49, 50, 53, 55, 59, 60] showed no significant differences in the WBC, LYM and CRP levels between two groups before intervention, respectively (P = 0.07, MD = − 0.07, 95% CI = − 0.14 to 0.00; P = 0.44, MD = 0.02, 95% CI = − 0.03 to 0.07; P = 0.50, MD = 0.16, 95% CI = − 0.31 to 0.63). However, after treatment, the level of WBC and LYM in combined CHM-WM group were significantly higher than WM group from the forest plot of eight trials [42, 43, 48, 50, 53, 55, 56, 58] related to WBC and six trials [42, 43, 48, 53, 55, 58] related to LYM (P = 0.005, MD = 0.61, 95% CI = 0.19–1.03; P = 0.0002, MD = 0.37, 95% CI = 0.17–0.56). Besides, fourteen trials [36, 40, 42–44, 48–50, 53, 55, 56, 58–60] reported that the CRP after treatment was significantly lower in combined CHM-WM group compared with WM group (P < 0.00001, MD = − 6.77, 95% CI = − 8.47 to − 5.07). Five trials [42, 43, 46, 50, 56] reported the LYM% before and after treatment was significantly higher in combined CHM-WM group than WM group (P < 0.00001, MD = 1.96, 95% CI = 1.30–2.62; P = 0.02, MD = 4.49, 95% CI = 0.73–8.26). The mean changes of PCT before treatment between two groups was assessed by six trials [36, 42, 46, 49, 55, 59] with no significant difference from the result of forest plot. However, after treatment, four trials [42, 49, 55, 59] reported significantly reduced of PCT in combined CHM-WM group compared with WM group (P = 0.06, MD = − 0.01, 95% CI = − 0.01 to 0.00; P = 0.01, MD = − 0.01, 95% CI = − 0.02 to − 0.00).

### Results on safety

**Fig. 3:**
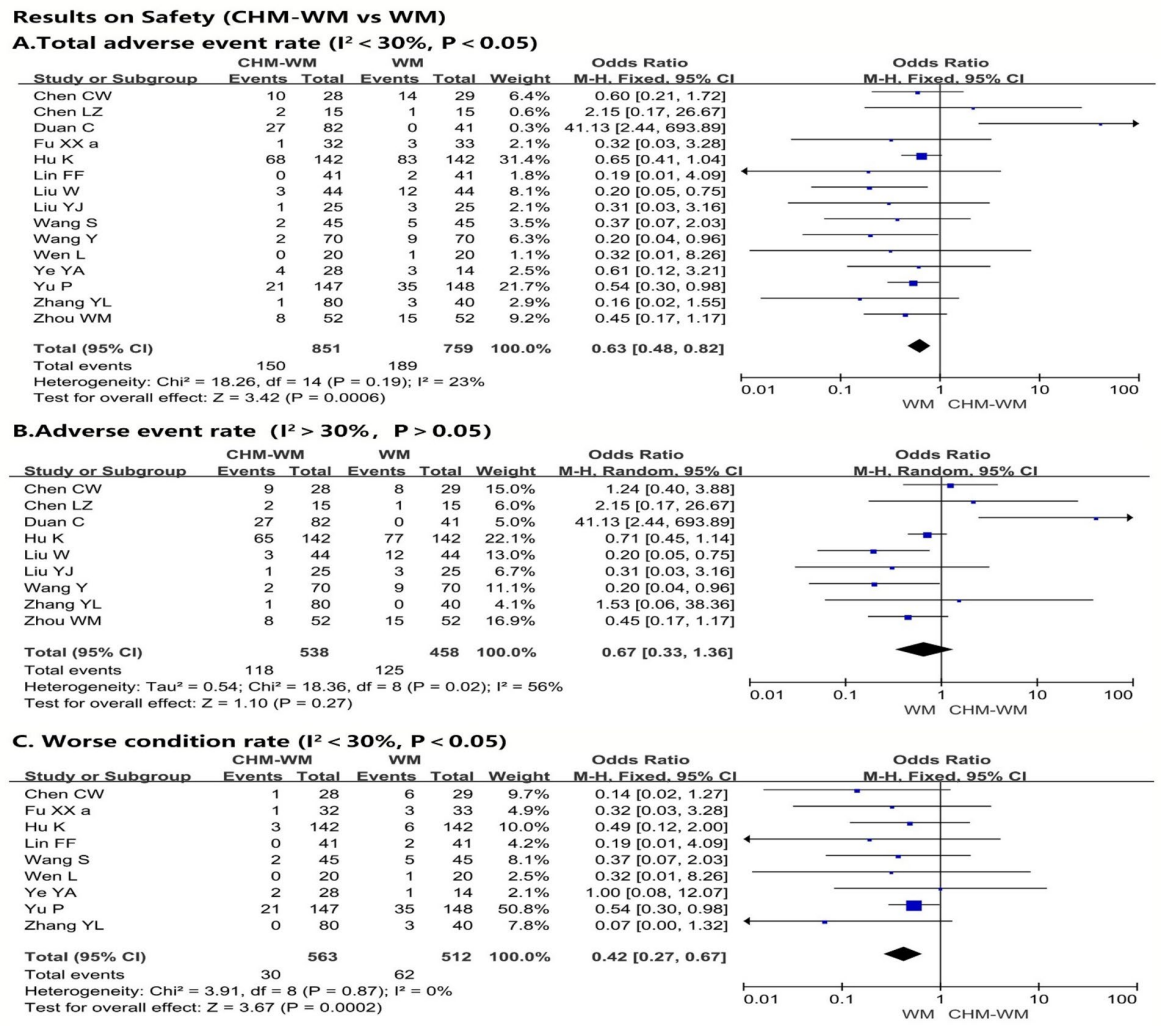
**A**–**C** Forest plot of the safety of combined CHM-WM vs WM

The total adverse event rate and worse condition rate during the treatment were reported by fifteen trials [36–42, 45–50, 53–55, 57, 60] and nine trials [36, 42, 45, 46, 49, 53, 55, 57], respectively, which increased significantly in WM group than combined CHM-WM group (P = 0.0006, OR = 0.63, 95% CI = 0.48 to 0.82, Fig. 3A; P = 0.0002, OR = 0.42, 95% CI = 0.27 to 0.67, Fig. 3C). However, there is no significant differences of adverse event rate between two groups according to the nine trials [36, 41, 45, 47, 48, 50, 57, 60] (P = 0.27, OR = 0.67, 95% CI = 0.33 to 1.36, Fig. 3B). Of the RCTs included, only one study reported one death in each group after treatment, and no serious adverse event was reported.

### Risk of bias assessment

**Fig. 4:**
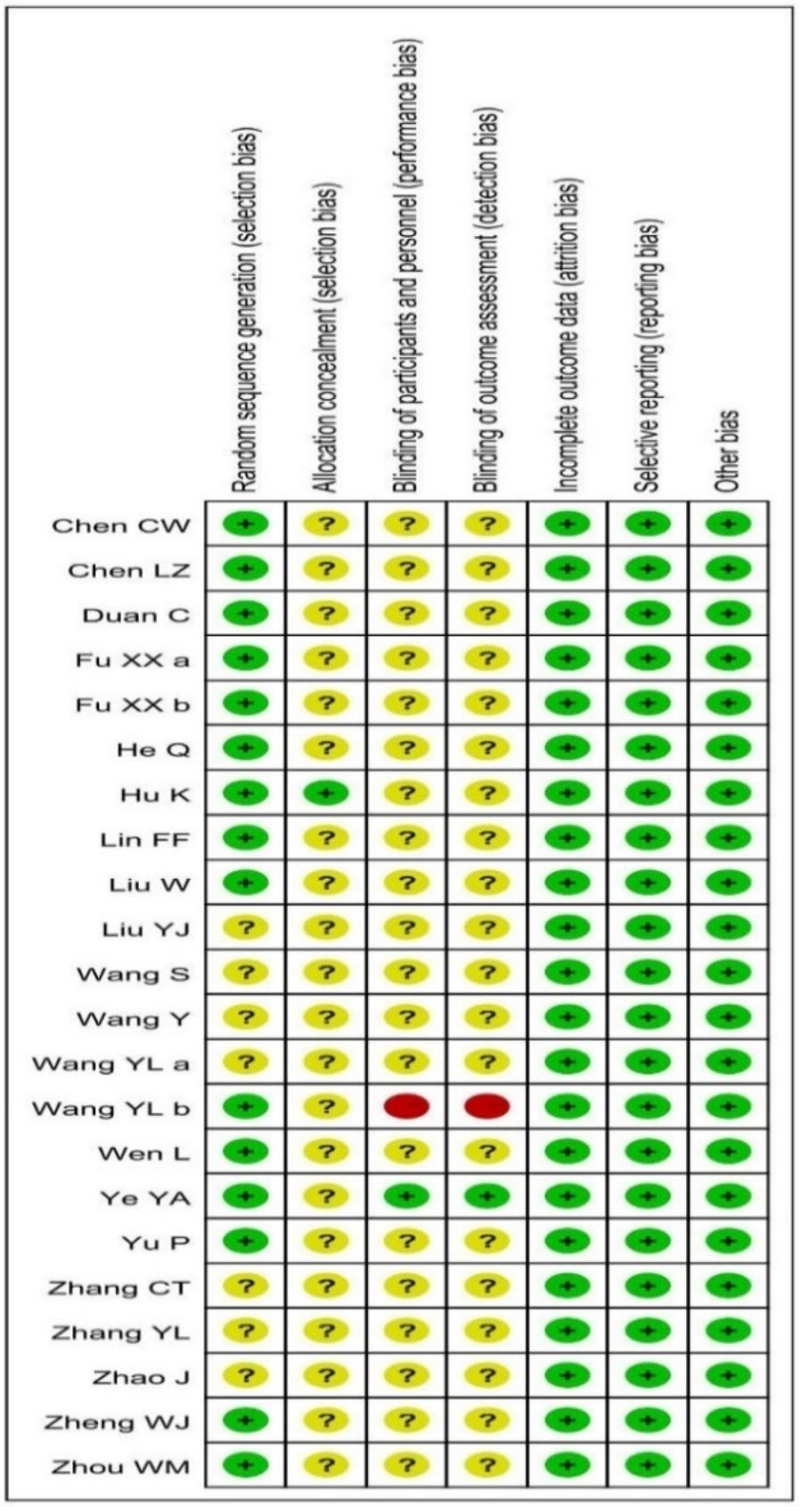
Summary on risk of bias

**Fig. 5:**
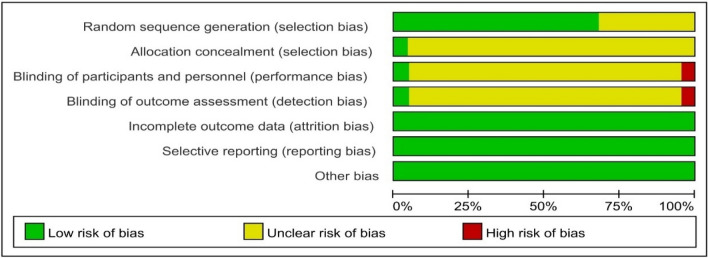
Risk of bias of included RCTs

The risks of the summaries on each bias were reported as shown in Fig. 4 and the bias of each included RCT with each intervention comparison were assessed as shown in Fig. 5. Sensitivity is not applicable since there is no high risk of bias in the allocation of participants to groups associated with a particular study or high levels of missing data.

### GRADE assessment

Different levels of quality for evidences were reported by GRADE assessment for combined CHM-WM vs WM (See Additional file 5). There were high evidences in total effectiveness rate, total adverse event rate and worse condition rate, moderate evidences in virological outcomes, CT improvement rate and adverse event rate, low to high evidences in symptom improvement and blood test improvement by GRADE assessment for combined CHM-WM vs WM treatment.

### Results on Subgroup

**Fig. 6:**
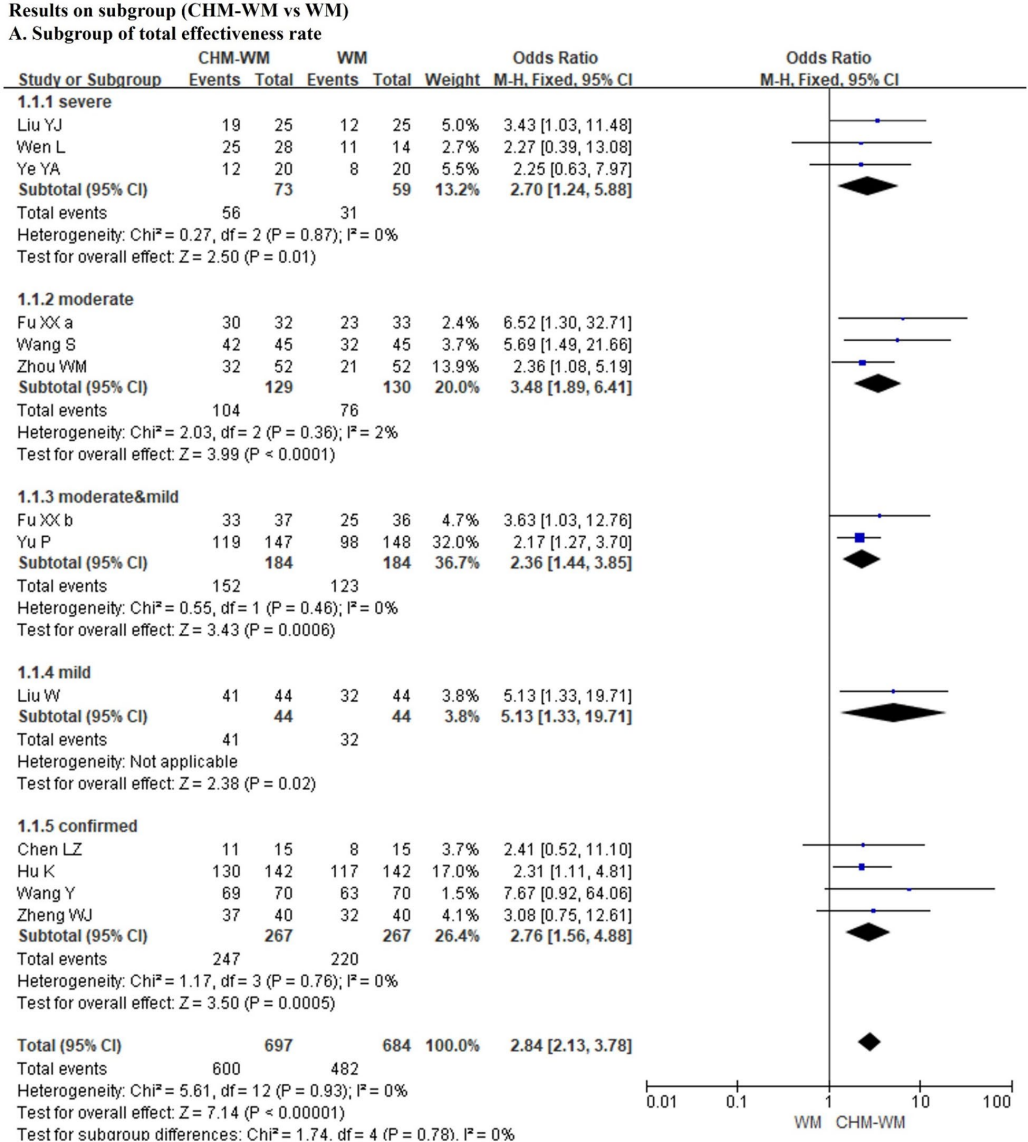

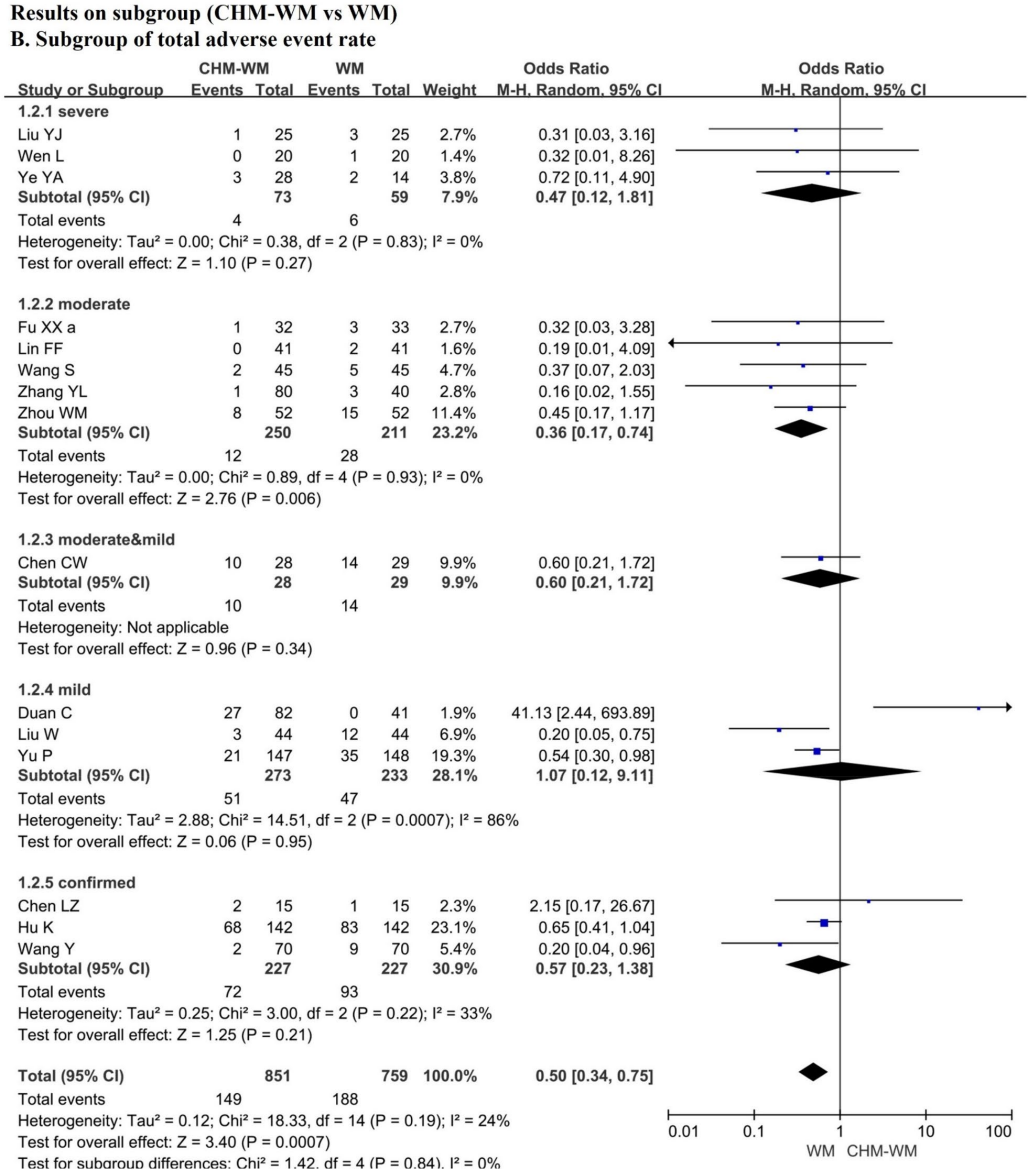
**A** Meta-analysis on subgroup of combined CHM-WM vs WM. **B** Meta-analysis on subgroup of combined CHM-WM vs WM

The results on different treatments based on different degrees of patients' conditions were reported in this study. There were thirteen trials [40, 42, 43, 45, 47, 50, 53, 55, 59, 60] reported the total effectiveness rate of subgroup increased significantly in combined CHM-WM group than WM group (P < 0.00001, OR = 2.84, 95%CI = 2.13 to 3.78, Fig. 6A). Of these, three trials related to severe patients [48, 53, 54], three trials [42, 49, 50] related to moderate patients, two trails [43, 55] related to moderate and mild patients, one trail [47] related to mild patients and four trails [40, 45, 50, 59] related to confirmed patients (P = 0.01, OR = 2.70, 95%CI = 1.24 to 5.88; P < 0.0001, OR = 3.48, 95%CI = 1.89 to 6.41; P = 0.0006, OR = 2.36, 95%CI = 1.44 to 3.85; P = 0.02, OR = 5.13, 95%CI = 1.33 to 19.71; P = 0.0005, OR = 2.76, 95%CI = 1.56 to 4.88). In addition, fifteen trails [36, 42, 45, 50, 53, 55, 57, 60] reported the adverse events of the subgroup based on different patients’ condition, which showed the total adverse event rate significant higher in WM group than the combined CHM-WM group (P = 0.0007, OR = 0.50, 95%CI = 0.34 to 0.75, Fig. 6B). Among these, three trials [48, 53, 54] related severe patients, five trials [42, 46, 49, 57, 60] related to moderate patients, one trial [36] related to moderate and mild patients, three trials [41, 47, 55] related to mild patients, three trials [40, 45, 50] related to confirmed patients (P = 0.27, OR = 0.47, 95%CI = 0.12 to 1.81; P = 0.006, OR = 0.36, 95%CI = 0.17 to 0.74; P = 0.34, OR = 0.60, 95%CI = 0.21 to 1.72; P = 0.95, OR = 1.07, 95%CI = 0.12 to 9.11; P = 0.21, OR = 0.57, 95%CI = 0.23 to 1.38). However, three RCTs of mild patient subgroups reported opposite findings on total adverse event rate. One author team reported combined CHM-WM caused more diarrhea as adverse event than WM treatment alone, and they concluded that it may due to patients’ intolerance to the high dose of CHM [41]. Whether diarrhea can be considered as an adverse reaction needs further study as it is reported that the SARS-CoV-2 was found in patients’ tools which implied that diarrhea could be a possible pathway to clear away the virus and relieve the patients’ condition [61, 62]. No trials used CHM alone compared with placebo or no treatment and no long-term outcomes were reported.

## Discussion

### Summary of evidence

In this review, 22 RCTs with good methodology investigating the efficacy of CHM for COVID-19 treatment were included. Comparing with WM, combined CHM-WM showed significant improvement in clinical, laboratory and radiographic index. Although there is no difference in mortality and adverse events in COVID-19 patients between the combined CHM-WM and WM groups, our findings implied that the CHM could be a potential therapy for COVID-19. GRADE approach showed the quality of evidence of the main index for efficacy and safety (total effectiveness rate and adverse events rate) are high, which implies that further study is very unlikely to change our current estimated better therapeutical effects of combined CHM-WM treatment compared to WM. Subgroup analysis of participants on different severity supported the efficacy and safety in the combined CHM-WM treatment, especially for mild and moderate patients.

Since its beginning, the COVID-19 has fast spread worldwide and caused a great number of people to death. Although clinical doctors and scientists acted speedily on all aspects for the diagnosis and treatment for the COVID-19 and over 300 clinical trials were registered nationally and internationally immediately, completed RCTs and valuable clinical data are still limited. Angiotensin converting enzyme II was considered as the target entry receptor of COVID-19, which may cause direct infection liver cell through fecal–oral transmission [63] In this review, Western medicines including remdesivir, lopinavir/ritonavir, favipiravir etc. were used to treat COVID-19 though regulate the function of liver and gastrointestinal [64]. However, adverse events were commonly reported during the treatment, for example, three published clinical studies on favipiravir or remdesivir in COVID-19 reported hepatotoxicity and digestive tract reaction, including nausea, vomit, diarrhea, abdominal pain [65–67].

Currently, no specific antiviral drug for COVID-19. Different from WM, CHM and its prescriptions have the characteristics of multi-component, multitargets and multipathways, and play an important role in broad-spectrum antiviral, anti-inflammatory, immune regulation and organ protection, which is commonly used for disease treatment in China [68, 69]. Studies showed CHM have great potential in preventing and treating COVID-19 by alleviating the "cytokine storm" and regulating Lung or respiratory system [70, 71]. Various Chinese patent medicine and Chinese herbal decoction (Table 3) were used in the included studies of this review, such as Lianhua Qingwen capsules, Jinhua Qingan granule, Xuanfei Baidu decoction, Xuebijing injection etc. Research showed CHM played a significant role in the fight against COVID-19 by improving immunity [72]. Network pharmacological strategy integrates molecular docking analyses indicated Lianhua Qingwen capsule can act by regulating immune response, apoptosis and virus infection, thereby exerting potential therapeutic effects in COVID-19. For the molecular mechanism of Lianhua Qingwen Capsule, the Akt1 was considered as the most important and promising drug target to reduce tissue damage and help to eliminate COVID-19 infection. In which, six active compounds of Lianhua Qiangwen capsule, namely beta-carotene, kaempferol, luteolin, naringenin, quercetin and wogonin showed the active potential with protein kinase B (AKT) [73]. Honeysuckle Flower (*Flos lonicerae*, Jin Yin Hua), Ephedra (*Herba Ephedrae*, Ma Huang), Pinellia tuber (*Pinelliae Rhizoma*, Ban Xia) and Bitter Apricot Seed (*Armeniacae Semen Amarum*, Ku Xing Ren) were the most commonly used CHM in the treatment of COVID-19. Research showed that the extract of Jinyinhua is a natural inhibitor of targeted AKT, which could inhabit the expression of PI3K/AKT inflammation pathway and significantly reduce the interleukin (IL)-1β, IL-6, tumor necrosis factor (TNF)-α and nuclear transcription factor kB (NF-kB), so as to effectively control the occurrence and development of inflammatory response [74]. In this review, comparing with WM, the adding of CHM not only improving the efficacy but also reducing adverse events to some extent, thus indicated CHM could be an alternative treatment in COVID-19.

**Table 3:** Components of Chinese herbal medicine and Western medicine used in the included studies

Refences	Treatments	Components	Western medicine	Components
	Chinese herbal medicine			
Chen 2021 [39]	Lianhua Qingwen Capsule	Weeping forsythia capsule (Lianqiao, *Fructus Forsythiae*), Honeysuckle bud and flower (Jinyinhua, *Flos Lonicerae*), Ephedra (Mahuang, *Herba Ephedrae*), Bitter apricot seed (Xingren, S*emen Armeniacae Amarum*), Gypsum (Shigao, *Gypsum Fibrosum*), Isatis root (Banlangen, *Radix Isatidis*), Male fern rhizome (Mianmaguanzhogn, *Rhizoma Dryopteris Crassirhizomae*), Heartleaf houttuynia herb (Yuxingcao, *Herba Houttuyniae*), Cablin patchouli herb (Guanghuoxiang, *Herba Pogostemonis*), Rhubarb root and rhizome (Dahuang, *Radix et Rhizoma Rhei*), Rose-boot (Hongjingtian, *Herba Rhodiolae*), Liquorice root (Gancao, *Radix Glycyrrhizae*)	Conventional antiviral drugs	%1Recombinant human interferon α2b, 5 million IU/time, add 2 mL of sterilized water for injection, inh, bid %1Lopinavir ritonavir tablets, po, bid
Chen 2020 [40]	Xuebijing Injection	Chinese angelica (Danggui, *Radix Angelicae Sinensis*), Safflower (Honghua, *Flos Carthami*), Danshen root (Danshen, *Radix Salviae Miltiorrhizae*), Sichuan lovage rhizome (Chuanxiong, *Rhizoma Ligustici Chuanxiong*), Peony root (Chishao, *Radix Paeoniae Rubra*)	Conventional antiviral drugs	Recombinant human interferon α2b, Lopinavir, Ritonavir
Duan 2020 [41]	Jinhua Qinggan granules	Honeysuckle bud and flower (Jinyinhua, *Flos Lonicerae*), Gypsum (Shigao, *Gypsum Fibrosum*), Ephedra (Mahuang, *Herba Ephedrae*), Bitter Apricot Seed (Kuxingren, *Semen Armeniacae Amarum*), Baical Skullcap Root (Huangqin, *Radix Scutellariae Baicalensis*), Forsythia Fruit (Lianqiao, *Fructus Forsythiae Suspensae*), Thunberbg Fritillary Bulb (Zhebeimu, *Bulbus Fritillariae Thunbergii*), Anemarrhena Rhizome (Zhimu, *Rhizoma Anemarrhenae Aspheloidis*), Great burdock achene (Niubangzi, *Fructus Arctii*), Sweet Wormwood (Qinghao, *Artemisiae Apiaceae seu Annuae Herba*), Field Mint (Bohe, *Herba Menthae Haplocalycis*), and Liquoric Root (Gancao, *Radix Glycyrrhizae*)	Conventional Western medicine	Based on National Health Commission of the People’s Republic of China and National Administration of Traditional Chinese Medicine jointly issued the ‘Diagnosis and Treatment Protocol for Novel Coronavirus Pneumonia’ (Trial Version 5), including antiviral and antiviral Infection and other symptomatic treatment, 5 days as a course of treatment
Fu 2020a [42]	Toujie Quwen granules	Forsythia Fruit (Lianqiao, *Fructus Forsythiae Suspensae*) 30 g, Appendiculate Cremastra Pseudobulb (Shancigu, *Pseudobulbus Cremastrae seu Pleiones)* 20 g, Honeysuckle bud and flower (Jinyinhua, *Flos Lonicerae*) 15 g, Baical Skullcap Root (Huangqin, *Radix Scutellariae Baicalensis*) 10 g, Dyers Woad Leaf (Daqingye, *Folium Isatidis*) 10 g, Thorowax Root (Chaihu, *Radix Bupleuri*) 5 g, Sweet Wormwood (Qinghao, *Artemisiae Apiaceae seu Annuae Herba*) 10 g, Circada Moulting (Chantui, *Periostracum Cicadae*) 10 g, Hogfennel Root (Qianhu, *Radix Peucedani*) 5 g, Tendrilleaf Fritillary Bulb (Chuanbeimu, *Bulbus Fritillariae Cirrhosae*) 10 g, Thunberbg Fritillary Bulb (Zhebeimu, *Bulbus Fritillariae Thunbergii*) 10 g, Smoked Plum (Wumei, *Fructus Mume*) 30 g, Ningpo Figwort Root (Xuanshen, *Radix Scrophulariae Ningpoensis*) 10 g, Astragalus (Huangqi, *Radix Astragali Membranacei*)45 g, Poria (Fuling, *Scierotium Poriae Cocos*)30 g, and Pseudostellaria Root (Taizishen, *Pseudostellariae Radix*) 15 g	Antiviral drugs	Abidor tablets, po, tid; Moxifloxacin tablets, po, qd; Ambroxol tablets, po, tid
Fu 2020b [43]	Toujie Quwen granules	Forsythia Fruit (Lianqiao, *Fructus Forsythiae Suspensae*) 30 g, Appendiculate Cremastra Pseudobulb (Shancigu, *Pseudobulbus Cremastrae seu Pleiones*) 20 g, Honeysuckle bud and flower (Jinyinhua, *Flos Lonicerae*) 15 g, Baical Skullcap Root (Huangqin, *Radix Scutellariae Baicalensis*) 10 g, Dyers Woad Leaf (Daqingye, *Folium Isatidis*) 10 g, Thorowax Root (Chaihu, *Radix Bupleuri*) 5 g, Sweet Wormwood (Qinghao, *Artemisiae Apiaceae seu Annuae Herba*) 10 g, Circada Moulting (Chantui, *Periostracum Cicadae*) 10 g, Hogfennel Root (Qianhu, *Radix Peucedani*) 5 g, Tendrilleaf Fritillary Bulb (Chuanbeimu, *Bulbus Fritillariae Cirrhosae*) 10 g, Thunberbg Fritillary Bulb (Zhebeimu, *Bulbus Fritillariae Thunbergii*) 10 g, Smoked Plum (Wumei, *Fructus Mume*) 30 g, Ningpo Figwort Root (Xuanshen, *Radix Scrophulariae Ningpoensis*) 10 g, Astragalus (Huangqi, *Radix Astragali Membranacei*) 45 g, Poria (Fuling, *Scierotium Poriae Cocos*)30 g, and Pseudostellaria Root (Taizishen, *Pseudostellariae Radix*) 15 g	Antiviral drugs	Abidor tablets, po, tid; Ambroxol tablets, po, tid
He 2021 [44]	Buzhong Yiqi decoction	Honey-scorched Astragalus (Huangqi, *Radix Astragali seu Hedysari*) 10 g, Panax ginseng (Renshen, *Radix Ginseng*) 3 g, Honey-fried liquorice root (Zhigancao, *Radix Glycyrrhizae*) 5 g, Fried atractylodes (Baizhu, *Rhizoma Atractylodis Macrocephalae*) 3 g, Dried tangerine peel (Chenpi, *Pericarpium Citri Reticulatae*) 3 g, Chinese angelica (Danggui, *Radix Angelicae Sinensis*) 3 g, Largetrifoliolious bugbane rhizome (Shengma, *Rhizoma Cimicifugae*) 3 g, Chinese thorowax root (Chiahu, *Radix Bupleuri*) 3 g	Antiviral drugs	Abidor tablets, 200 mg, po, tid
Hu 2021 [45]	Lianhua Qingwen capsules	Forsythia Fruit (Lianqiao, *Fructus Forsythiae Suspensae*), Honeysuckle bud and flower (Jinyinhua, *Flos Lonicerae*), Ephedra (Mahuang, *Herba Ephedrae*), Bitter Apricot Seed (Kuxingren, *Semen Armeniacae Amarum*), Gypsum (Shigao, *Gypsum Fibrosum*), Indigowoad Root (Banlangen, *Radix Isatidis*), Male fern rhizome (Mianma Guanzhong, *Rhizoma Dryopteris Crassirhizomae*), Heartleaf houttuynia herb (Yuxingcao, *Herba Houttuyniae*), Cablin patchouli herb (Huoxiang, *Herba Pogostemonis*), Rhubarb Root and Rhizome (Dahuang, *Radix Et Rhizoma Rhei*), Rose-boot (Hongjingtian, *Herba Rhodiolae*), menthol, and Liquoric Root (Gancao, *Radix Glycyrrhizae*)	Routine treatment	Oxygen therapy, antiviral medications and symptomatic therapies
Lin 2020 [46]	Xuanfeiqingre decoction	Ephedra (Mahuang, *Herba Ephedrae*) 9 g, Bitter apricot seed (Xingren, *Semen Armeniacae Amarum*) 12 g, Gypsum (Shigao, *Gypsum Fibrosum*) 30 g, Liquorice root (Gancao, *Radix Glycyrrhizae*) 6 g, Peach seed (Taoren, *Semen Persicae*) 12 g, Winter melon kernels (Dongguaren, *Benincasa hispida Cogn*) 30 g, Reed rhizome (Lugen, *Rhizoma Phragmitis*) 30 g, Coix seed (Yiyiren, *Semen Coicis*) 30 g, Platycodon root (Jiegeng, *Radix Platycodonis*) 9 g, Ginger processed pinellia (Jiangbanxia, *Rhizome Pinelliae Preparata*) 12 g, Longstamen onion bulb (Xiebai, *Bulbus Allii Macrostemonis*) 12 g, Fruit of caoguo (Caoguo, *Fructus Tsaoko*) 6 g, Cablin patchouli herb (Huoxiang, *Herba Pogostemonis*) 10 g	Conventional treatment in Western medicine	α-interferon, Lopinavir/ritonavir tablets
Liu 2021 [47]	Lianhuaqingwen capsules, Pneumonia 2 defined formula	Pneumonia 2 defined formula: Bitter apricot seed (Xingren, *Semen Armeniacae Amarum*), Ephedra (Mahuang, *Herba Ephedrae*), Ginkgo seed (Baiguo, *Semen Ginkgo*), Earthworm (Dilong, *Lumbricus*), Pepperweed seed (Tinglizi, *Semen Descurainiae*), Chinese magnoliavine fruit (Wuweizi, *Fructus Schisandrae Chinensis*), Pinellia tuber (Banxia, *Rhizoma Pinelliae*), Liquorice root (Gancao, *Radix Glycyrrhizae*), Perilla fruit (Zisuzi, *Fructus Perillae*), White mulberry root-bark (Sang, Baipi, *Cortex Mori*), Common coltsfoot flower (Kuandonghau, *Flos Farfarae*)	Antiviral drugs	Abidord tablets, Oseltamivir
Liu 2021 [48]	Huashi Baidu formula	Ephedra (Mahuang, Herba Ephedrae) 6 g, Bitter apricot seed (Xingren, Semen Armeniacae Amarum) 9 g, Gypsum (Shigao, Gypsum Fibrosum) 15 g, Liquorice root (Gancao, Radix Glycyrrhizae) 3 g, Cablin patchouli herb (Guanghuoxiang, Herba Pogostemonis) 10 g, Officinal magnolia bark (Houpu, Cortex Magnoliae Officinalis) 10 g, Atractylodes rhizome (Cangzhu, Rhizoma Atractylodis) 15 g, Fruit of caoguo (Caoguo, Fructus Tsaoko) 10 g, Processed pinellia tuber (Fabanxia, Rhizoma Pinelliae Preparatum) 9 g, Indian bread (Fuling, Poria) 15 g, Rhubarb root and rhizome (Dahuang, Radix et Rhizoma Rhei) 5 g, Milkvetch root (Huangqi, Radix Astragali seu Hedysari) 10 g, Pepperweed seed (Tinglizi, Semen Descurainiae) 10 g, Peony root (Chishao, Radix Paeoniae Rubra) 10 g	Western medicine treatment	Tocilizumab, oxygen therapy, mechanical ventilation, plasma therapy for convalescent patients
Wang 2020 [49]	Viable Bifidobacterium Tablets combined with Sanren decoction	Sanren decoction: Processed pinellia tuber (Fabanxia, *Rhizoma Pinelliae Preparatum*) 9 g, Lophatherum herb (Danzhuye, *Herba Lophatheri*) 24 g, Ricepaperplant pith (Tongcao, *Medulla Tetrapanacis*) 3 g, Talc (Huashi, *Talcum*) 18 g, Bitter apricot seed (Xingren, *Semen Armeniacae Amarum*) 12 g, Coix seed (Yiyiren, *Semen Coicis*) 45 g, Chinese thorowax root (Chaihu, *Radix Bupleuri*) 6 g, Baical skullcap root (Huangqin, *Radix Scutellariae*) 24 g, Heterophylly falsestarwort root (Taizishen, *Radix Pseudostellariae*) 24 g, Turmeric root tuber (Yujin, *Radix Curcumae*) 24 g, Indian bread (Fuling, Poria) 45 g, Finger citron (Foshou, *Fructus Citri Sarcodactylis*) 24 g, Reed rhizome (Lugen, *Rhizoma Phragmitis*) 45 g	Antiviral drugs	Oseltamivir capsules, Ambroxol tablets
Wang 2021 [50]	Qingfei Paidu decoction	Honey-fried liquorice root (Zhigancao, *Radix Glycyrrhizae*) 6 g, Ephedra (Mahuang, *Herba Ephedrae*) 9 g, Gypsum (Shengshigao, *Gypsum Fibrosum*) 15 ~ 30 g, Bitter apricot seed (Xingren, *Semen Armeniacae Amarum*), Grifola (Zhuling, *Polyporus Umbellatus*) 9 g, Cassia twig (Guizhi, *Ramulus Cinnamomi*) 9 g, Largehead atractylodes rhizome (Baizhu, *Rhizoma Atractylodis Macrocephalae*) 9 g, Oriental waterplantain rhizome (Zexie, *Rhizoma Alismatis*) 9 g, Chinese thorowax root (Chaihu, *Radix Bupleuri*) 16 g, Indian bread (Fuling, *Poria*) 15 g, Baical skullcap root (Huangqin, *Radix Scutellariae*) 6 g, Blackberry lily rhizome (Shegan, *Rhizoma Belamcandae*) 9 g, Ginger processed pinellia (Jiangbanxia, *Rhizome Pinelliae Preparata*) 9 g, Tatarian Aster Root (Ziyuan, *Aster tataricus*) 9 g, Fresh ginger (Shengjiang, *Rhizoma Zingiberis Recens*) 9 g, Cablin patchouli herb (Huoxiang, *Herba Pogostemonis*) 9 g, Immature orange fruit (Zhishi, *Fructus Aurantii Immaturus*) 6 g, Dried tangerine peel (Chenpi, *Pericarpium Citri Reticulatae*) 6 g, Manchurian wildginger (Xixin, *Herba Asari*) 6 g, Common yam rhizome (Shanyao, R*hizoma Dioscoreae*) 12 g, Common coltsfoot flower (Kuandonghua, *Flos Farfarae*) 9 g	Routine treatment	Moxifloxacin hydrochloride tablets, Arbidol hydrochloride dispersible tablets
Wang 2020a [51]	Chinese medicinal formulae	Buzhongyiqi supplemented formula: Milkvetch root (Huangqi, *Radix Astragali seu Hedysari*) 30 g, Ginseng (Renshen, *Radix Ginseng*) 15 g, Liquorice root (Gancao, *Radix Glycyrrhizae*) 15 g, Largehead atractylodes rhizome (Baizhu, *Rhizoma Atractylodis Macrocephalae*) 10 g, Dried tangerine peel (Chenpi, *Pericarpium Citri Reticulatae*) 6 g, Chinese angelica (Danggui, *Radix Angelicae Sinensis*) 10 g, Chinese date (Dazao, *Fructus Jujubae*) 6 pieces, Fresh ginger (Shengjiang, *Rhizoma Zingiberis Recens*) 9 pieces, Chinese thorowax root (Chaihu, *Radix Bupleuri*) 12 g, Largetrifoliolious bugbane rhizome (Shengma, *Rhizoma Cimicifugae*) 6 g Huhuang Paidu supplemented formula: Golden thread (Huanglian, *Rhizoma Coptidis*) 20 g, Rhubarb root and rhizome (Shengdahuang, *Radix et Rhizoma Rhei*) 10 g, Baical skullcap root (Shenghuangqin, *Radix Scutellariae*) 10 g, Tatarian Aster Root (Ziyuan, *Aster tataricus*) 10 g, Heartleaf houttuynia herb (Yuxingcao, *Herba Houttuyniae*) 10 g, Dandelion (Pugongying, *Herba Taraxaci*) 10 g, Giant knotweed rhizome (Huzhang, *Rhizoma Polygoni Cuspidati*) 10 g, Milkvetch root (Huangqi, *Radix Astragali seu Hedysari*) 20 g Baimu Qingre Jiedu formula: Kudzuvine root (Gegen, *Radix Puerariae*) 15 g, Dahurian angelica root (Baizhi, *Radix Angelicae Dahuricae*) 12 g, Biond magnolia flower-bud (Xinyi, *Flos Magnoliae*) 9 g, Isatis root (Banlangen, *Radix Isatidis*) 30 g, Weeping forsythia capsule (Lianqiao, *Fructus Forsythiae*) 15 g, Thunberbg fritillary bulb (Zhebeimu, *Bulbus Fritillariae Thunbergii*) 12 g Herbal fumigating ointment: Golden thread (Huanglian, *Rhizoma Coptidis*) 20 g, Rhubarb root and rhizome (Dahuang, *Radix et Rhizoma Rhei*) 10 g, Baical skullcap root (Huangqin, *Radix Scutellariae*) 10 g, Atractylodes rhizome (Cangzhu, *Rhizoma Atractylodis*) 10 g, Tatarian Aster Root (Ziyuan, *Aster tataricus*) 10 g, Heartleaf houttuynia herb (Yuxingcao, *Herba Houttuyniae*) 10 g, Dandelion (Pugongying, *Herba Taraxaci*) 10 g, Giant knotweed rhizome (Huzhang, *Rhizoma Polygoni Cuspidati*) 10 g, Milkvetch root (Huangqi, *Radix Astragali seu Hedysari*) 20 g Vitamin C, Vitamin E, folic acid	Antiviral drugs	Ribavirin, anti-infective and adjunctive supportive medications
Wang 2020b [52]	Qingre Kangdu Oral liquid	Gypsum (Shengshigao, *Gypsum Fibrosum*), Isatis root (Banlangen, *Radix Isatidis*), Largetrifoliolious bugbane rhizome (Shengma, *Rhizoma Cimicifugae*), Cablin patchouli herb (Guanghuoxiang, *Herba Pogostemonis*), Dandelion (Pugongying, *Herba Taraxaci*), Figwortflower picrorhiza rhizome (Huanglian, *Rhizoma Picrorhizae*), Tree peony root bark (Mudanpi, *Cortex Moutan Radicis*), Cogon grass rhizome (Baimaogen, *Rhizoma Imperatae*), Mung bean (Lvdou, *Viginal radiate*)	Antiviral drugs	Recombinant human interferon α2b, Arbidol hydrochloride tablets
Wen 2020 [53]	Xuebijing injection	Safflower (Honghua, *Flos Carthami*), Peony root (Chishao, *Radix Paeoniae Rubra*), Sichuan lovage rhizome (Chuanxiong, *Rhizoma Ligustici Chuanxiong*), Chinese angelica (Danggui, *Radix Angelicae Sinensis*)	Conventional treatment	Follow the COVID-19 diagnosis and treatment guidelines issued by the National Health Commission (NHC)
Ye 2020 [54]	CHM foemula	NR	Routine pharmaceutical medications	Ribavirin/arbidole tablets
Yu 2020 [55]	Lianhua Qingwen granules	Forsythia Fruit (Lianqiao, *Fructus Forsythiae Suspensae*), Honeysuckle bud and flower (Jinyinhua, *Flos Lonicerae*), Ephedra (Mahuang, *Herba Ephedrae*), Bitter Apricot Seed (Kuxingren, *Semen Armeniacae Amarum*), Gypsum (Shigao, *Gypsum Fibrosum*), Indigowoad Root (Banlangen, *Radix Isatidis*), Male fern rhizome (Mianma Guanzhong, *Rhizoma Dryopteris Crassirhizomae*), Heartleaf houttuynia herb (Yuxingcao, *Herba Houttuyniae*), Cablin patchouli herb (Huoxiang, *Herba Pogostemonis*), Rhubarb Root and Rhizome (Dahuang, *Radix Et Rhizoma Rhei*), Rose-boot (Hongjingtian, *Herba Rhodiolae*), menthol, and Liquoric Root (Gancao, *Radix Glycyrrhizae*)	Antiviral drugs	Arbidol hydrochloride dispersible tablets, po, tid; Moxifloxacin tablets, po, qd; Ambroxol tablets, po, tid
Zhang 2020 [56]	Jiaweida formula	Honey-fried ephedra (Zhimahuang, *Herba Ephedrae*) 10 g, Bitter apricot seed (Xingren, *Semen Armeniacae Amarum*) 15 g, Gypsum (Shengshigao, *Gypsum Fibrosum*) 20 g, Snakegourd peel (Gualoupi, *Pericarpium Trichosanthis*) 20 g, Rhubarb root and rhizome (Dahuang, *Radix et Rhizoma Rhei*) 6 g, Pepperweed seed (Tinglizi, *Semen Descurainiae*) 10 g, Peach seed (Taoren, *Semen Persicae*), Fruit of caoguo (Caoguo, *Fructus Tsaoko*) 6 g, Areca seed (Binglang, *Semen Arecae*) 10 g, Atractylodes rhizome (Cangzhu, *Rhizoma Atractylodis*) 10 g	Basic treatment	Oxygen therapy, antiviral medications and symptomatic therapies
Zhang 2020 [57]	Jinyinhua oral liquid	Honeysuckle bud and flower (Jinyinhua, *Flos Lonicerae*)	Antiviral drugs	α-interferon, Lopinavir/ritonavir tablets
Zhao 2020 [58]	CM prescription	Bitter apricot seed (Xingren, *Semen Armeniacae Amarum*) 10 g, Calcium sulfate dehydrate (Shengshigao, *Gypsum Fibrosum*) 30 g, Snakegourd fruit (Gualou, *Fructus Trichosanthis*) 30 g, Rhubarb root and rhizome (Dahuang, *Radix et Rhizoma Rhei*) 6 g, Ephedra (Mahuang, *Herba Ephedrae*) 6 g, Lepidium apetalum Willd (Tinglizi, *Semen Descurainiae*) 10 g, Peach seed (Taoren, *Semen Persicae*) 10 g, Fruit of caoguo (Caoguo, *Fructus Tsaoko*) 6 g, Areca seed (Binglang, *Semen Arecae*) 10 g, Atractylodes rhizome (Cangzhu, *Rhizoma Atractylodis*) 10 g	General strategies	The general strategies were according to the National recommendations for diagnosis and treatment of pneumonia caused by SARS-COV-2 (the 5th edition), including bed rest and supportive treatments; ensuring sufficient calories and water intake; maintaining water electrolyte balance and homeostasis, and strengthening psychotherapy for elder children when necessary
Zheng 2020 [59]	Tanshinone II A Sodium Sulfonate	NR	Isolation and symptomatic supportive care	It includes oxygen inhalation, fluid rehydration, anti-inflammatory and disease resistance as well as ventilator assisted breathing
Zhou 2020 [60]	Enteric-soluble capsule of diamine glycyrrhizinate	NR	Routine treatment	Strengthen support treatment, maintain water and electrolyte balance, maintain internal environment stability, effective oxygen therapy; Lopinavir/ritonavir tablets

*Qd* Once a day, *Bid* Twice a day, *Tid* Three times a day, *Po* Oral administration

### Advances compared with prior systematic review

Since the outbreak of COVID-19, some systematic review and meta-analysis were conducted to assess the efficacy and safety of CHM for COVID-19. However, most of the published reviews were limited to small number of RCTs (from 6 to 7) [75–77], small sample size in each RCT [75–78], low certainty evidence on main outcomes [75], poor methodology design including obvious high risk of bias in selective reporting and incomplete outcomes [75, 76]. Compared with the published reviews, our review included larger number of RCTs (n = 22) with high certainty evidence and low risk of bias on primary outcomes and various types of CHM, which provided more comprehensive outcome index and increased credibility. Based on literature search, this is the first time to assess the efficacy and safety of combined CHM-WM for different severity participants on COVID-19, which can provide guidance in rational use of CHM for further clinical trials.

### Limitation

Limitations in this review should also be taken into consideration as follows. Firstly, according to the unique diagnosis and classification of Chinese medicine, the formulae may differ due to the type and severity of patients. Most Chinese medicine practitioners would slightly modify the classical prescriptions depending on the individual clinical presentation. Some CHMs have been added into or removed out of the classical formula during treatment. Therefore, the conclusion on total effectiveness rate in our study could only be in general terms and not referring to any individual CHM or specific formula. Secondly, some meta-analysis results were still with heterogeneity limitation even a random model was applied to those analyses with I^2^ > 30% in the fixed model, which may more or less affect the strength of evidence. Finally, according to the GRADE handbook from Cochrane website, the evaluation on evidence was influenced by many aspects, including study design, study limitations (risk of bias including random sequence generation, allocation concealment, blinding of participants and personnel, blinding of outcome assessment, incomplete outcome data, selective reporting and other bias), inconsistency of results, indirectness of evidence, imprecision, publication bias etc. Therefore, only one high risk of blinding bias in one included RCT [52] did not affect our final conclusion on evidence level.

### Implications for clinical practice and research

The findings of our study supported combined CHM-WM could be a better alternative therapeutical method as a treatment for COVID-19 compared with WM. The existing evidence in this review might help to improve the design of future trials and guide clinicians in syndrome differentiation and treatment. Although we have discussed the possible mechanism of CHM in COVID-19 treatment, there is still have much scope and great significance for further explore the pharmaceutical properties and antiviral mechanisms of the various ingredients in CHM on COVID-19 treatment. Since the COVID-19 epidemic has not completely subsided, no effective treatment protocols and evidence from current studies are incomplete, thus, more RCTs with multicenter, large-sample and strict methodology are needed to further complete the effectiveness and safety of combined CHM-WM for COVID-19.

## Conclusion

The results of the current meta-analysis supported combined CHM-WM could be as potential candidates in COVID-19 treatment, especially patients with mild and moderate symptoms. According to the findings, combined CHM-WM exhibited superior performance in clinical symptoms, blood test and virological outcome improvement compared with WM. In terms of limitations, large sample and multicenter RCTs are important and worthy in further study to further confirm the effectiveness and adverse events of CHM in the treatment of COVID-19.

## Supplementary Information

**Additional file 1**. Search strategy for English Databases.

**Additional file 2**. Search Strategies for Chinese Databases.

**Additional file 3**. INPLASY protocol.

**Additional file 4**. PRISMA checklist 2020.

**Additional file 5**. GRADE assessment for combined CHM-WM vs WM treatment.

## Data Availability

Not applicable.

## Abbreviations

CHM: Chinese herbal medicine
COVID-19: Coronavirus disease 2019
WM: Western Medicine
RCT: Randomized clinical trial
CT: Computerized tomography
SARS-CoV-2: Severe acute respiratory syndrome coronavirus-2
α-INF: Alpha interferon
WBC: White blood cell count
LYM: Lymphocyte absolute value
LYM%: Lymphocyte ratio
CRP: C-reactive protein
PCT: Procalcitonin
SAE: Serious adverse event cases
NCP: Novel coronavirus pneumonia
PaO_2_: Arterial partial pressure of oxygen
FiO_2_: Fraction of inspired oxygen
RR: Risk ratio
BPM: Beat per minute
RT-PCR: Reverse transcription-polymerase chain reaction
OR: Odds ratio
CI: Confidence interval
MD: Mean difference
GRADE: Grades of recommendations assessment, development and evaluation
Qd: Once a day
Bid: Twice a day
Tid: Three times a day
Qid: Four times a day
NR: Not reported
TCM: Traditional Chinese medicine
IL-10: Interleukin-10
IL-6: Interleukin-6
IL-4: Interleukin-4
AKT: Protein kinase B
NF-kB: Nuclear transcription factor kB
hs-CRP: Hypersensitive-c-reactive-protein
NEU: Neutrophils
ALT: Alaninetransaminase
AST: Glutamic oxaloacetic transaminase
ESR: Erythrocyte sedimentation rate
APACHE-II: Acute physiology and chronic health evaluation
CD: Cluster of differentiation
TNF-α: Tumor necrosis factor
