# Supplementary material for: The efficacy and safety of combined chinese herbal medicine and western medicine therapy for COVID-19: a systematic review and meta-analysis
Source: Chin Med. 2022 Jun 21;17:77. doi: 10.1186/s13020-022-00600-z (PMC9210065; doi:10.1186/s13020-022-00600-z)
Supplement: Supplementary file 1 — Additional file 1. Search strategy for English Databases. [file 13020_2022_600_MOESM1_ESM.docx]

**Additional files**

| **Serial Number** | | **Strategy** |
| --- | --- | --- |
| **PubMed** | |  |
| #1 | "severe acute respiratory syndrome coronavirus 2" [Supplementary Concept] | |
| #2 | "COVID-19" [Supplementary Concept] | |
| #3 | "novel coronavirus pneumonia"[Title/Abstract] OR "2019-nCoV"[Title/Abstract] OR "COVID-19"[Title/Abstract] OR "COVID-2019"[Title/Abstract] OR "SARS-CoV-2" [Title/Abstract] OR "NCP" [Title/Abstract] OR "coronavirus disease-19"[Title/Abstract] OR "COVID19"[Title/Abstract] OR "coronavirus disease-19"[Title/Abstract] | |
| #4 | OR/1-3 | |
| #5 | "Clinical trial"[Title/Abstract] OR"Randomized clinical control"[Title/Abstract] | |
| #6 | "2020/01/01"[Date - Create] : "2021**/**06/30"[Date - Create] | |
| #7 | #4 AND #5AND #6 | |
|  |  | |
| **Embase** | | |
| #1 | (''Novel coronavirus pneumonia'':ti,ab,kw OR ''2019-ncov'':ti,ab,kw OR ''COVID-19'':ti,ab,kw OR ''Covid-2019'':ti,ab,kw OR ''Sars-cov-2'':ti,ab,kw OR ''NCP'':ti,ab,kw OR ''Covid19'':ti,ab,kw OR ''Coronavirus disease-19'':ti,ab,kw) | |
| #2 | ''Randomized clinical control'':ti,ab,kw | |
| #3 | Date 01-01-2020 to 30-06-2021 | |
| #4 | #1 AND # 2 AND #3 | |
|  |  | |
| **ICTRP** | | |
| #1 | ("COVID-19"OR"2019-nCoV"OR"SARS-CoV-2"OR"coronavirus disease 2019" OR "novel coronavirus pneumonia" OR "NCP"):ti,ab,kw | |
| #2 | "Randomized clinical control'':ti,ab,kw | |
| #3 | #1 AND # 2 | |
| **ClinicalTrials.gov** | | |
| #1 | ("COVID-19"OR"2019-nCoV"OR"SARS-CoV-2"OR"coronavirus disease 2019" OR "novel coronavirus pneumonia" OR "NCP"):ti,ab,kw | |
| #2 | "Randomized clinical control'':ti,ab,kw | |
| #3 | #1 AND # 2 | |

**Additional file 1.** **Search strategy for English Databases**
