# Supplementary material for: The efficacy and safety of combined chinese herbal medicine and western medicine therapy for COVID-19: a systematic review and meta-analysis
Source: Chin Med. 2022 Jun 21;17:77. doi: 10.1186/s13020-022-00600-z (PMC9210065; doi:10.1186/s13020-022-00600-z)
Supplement: Supplementary file 2 — Additional file 2. Search Strategies for Chinese Databases. [file 13020_2022_600_MOESM2_ESM.docx]

**Additional file 2.** **Search Strategies for Chinese Databases**

We used a similar search strategy for China National Knowledge Infrastructure (CNKI); China Chinese Clinical Trial Registry (ChiCTR) and Wanfang Database. The search key words included “COVID-19”, “RCT”, “randomized clinical trial*”, “Chinese herbal medicine*”, “Chinese medicine*”, “integrative treatment*”, “severity” and their relevant wordings.
