# Supplementary material for: The efficacy and safety of combined chinese herbal medicine and western medicine therapy for COVID-19: a systematic review and meta-analysis
Source: Chin Med. 2022 Jun 21;17:77. doi: 10.1186/s13020-022-00600-z (PMC9210065; doi:10.1186/s13020-022-00600-z)
Supplement: Supplementary file 3 — Additional file 3. INPLASY protocol. [file 13020_2022_600_MOESM3_ESM.pdf]

INPLASY
International Platform of Registered Systematic Review and Meta-analysis Protocols

INPLASY202210072

The Efficacy and Safety of Combined Chinese Herbal Medicine and Western Medicine Therapy for COVID-19: A Systematic Review and Meta-analysis

INPLASY PROTOCOL

To cite: Li et al. The Efficacy and Safety of Combined Chinese Herbal Medicine and Western Medicine Therapy for COVID-19: A Systematic Review and Meta-analysis. Inplasy protocol 202210072. doi: 10.37766/inplasy2022.1.0072

Received: 15 January 2022

Published: 15 January 2022

Corresponding author:
Lu Li

luciali@zju.edu.cn

Author Affiliation:
Pharmaceutical Informatics Institute, College of Pharmaceutical Sciences, Zhejiang University.

Support: **LY20H180004, 2020-KYGG-01-04.**

Review Stage at time of this submission: **Completed but not published.**

Conflicts of interest:
None declared.

The Efficacy and Safety of Combined Chinese Herbal Medicine and Western Medicine Therapy for COVID-19: A Systematic Review and Meta-analysis

Li, L[1]; Xie, HL[2]; Wang, L[3]; Zhang, AL[4]; Mou, X[5]; Lin, YF[6]; Ma, HL[7]; Wang, Y[8]; Li, J[8]; Gao, JS[10]; Wang, CC[11]; Leung, CP[12]; Fan, XH[13]; Wu, XK[4].

Review question / Objective: **To systematically review the clinical efficacy and safety of Chinese herbal medicine (CHM) with and without Western medicine (WM) for different severity of COVID-19.**
Condition being studied: **Since its beginning, the COVID-19 has fast spread worldwide and caused a great number of people to death. Although clinical doctors and scientists acted speedily on all aspects for the diagnosis and treatment for the COVID-19 and over 300 clinical trials were registered nationally and internationally immediately, completed RCTs and valuable clinical data are still limited. Currently, no specific antiviral drugs or efficient vaccines are available to prevent or treat COVID-19 infection, symptomatic and supportive treatments are still the mainstream strategies to manage the infection in clinical practice. Compared with Western medicine alone, combined Cheses herbal medicine and Western medicine exhibited remarkable benefits against the prevention, treatment and rehabilitation of COVID-19. Several systematic reviews on the efficacy of CHM for the treatment of COVID-19 have been published, however, their deficiency in methodological have limited their clinical guidance and increased potential bias. Besides, there is a lack of evidence to support the efficacy and safety of combined CHM-WM for different severity participants. Therefore, a comprehensive and rigorous evaluation of clinical research using combined CHM-WM for COVID-19 is needed.**

INPLASY registration number: This protocol was registered with the International Platform of Registered Systematic Review and Meta-Analysis Protocols (INPLASY) on 15 January 2022 and was last updated on 15 January 2022 (registration number INPLASY202210072).

INTRODUCTION

Review question / Objective: **To systematically review the clinical efficacy and safety of Chinese herbal medicine (CHM) with and without Western medicine (WM) for different severity of COVID-19.**

Li et al. Inplasy protocol 202210072. doi:10.37766/inplasy2022.1.0072 Downloaded from https://inplasy.com/inplasy-2022-1-0072/

Condition being studied: Since its beginning, the COVID-19 has fast spread worldwide and caused a great number of people to death. Although clinical doctors and scientists acted speedily on all aspects for the diagnosis and treatment for the COVID-19 and over 300 clinical trials were registered nationally and internationally immediately, completed RCTs and valuable clinical data are still limited. Currently, no specific antiviral drugs or efficient vaccines are available to prevent or treat COVID-19 infection, symptomatic and supportive treatments are still the mainstream strategies to manage the infection in clinical practice. Compared with Western medicine alone, combined Cheses herbal medicine and Western medicine exhibited remarkable benefits against the prevention, treatment and rehabilitation of COVID-19. Several systematic reviews on the efficacy of CHM for the treatment of COVID-19 have been published, however, their deficiency in methodological have limited their clinical guidance and increased potential bias. Besides, there is a lack of evidence to support the efficacy and safety of combined CHM-WM for different severity participants. Therefore, a comprehensive and rigorous evaluation of clinical research using combined CHM-WM for COVID-19 is needed.

METHODS

Participant or population: Patients diagnosed with COVID-19 infection, regardless of age, gender, nationality, duration of sickness, severity, etc.

Intervention: Chinese herbal medicine alone or combined Chinese herbal medicine with Western medicine were included.

Comparator: Western medicine, placebo, no treatment and standard care were included as control.

Study designs to be included: Only randomized clinical trials were included

Eligibility criteria: (1) All patients diagnosed with COVID-19 or tested positive were studied, regardless of age, gender, nationality, duration of sickness and severity, etc. (2)Only RCTs comparing CHM treatment with placebo, no or other treatment for COVID-19 patients were eligible for inclusion. (3) We excluded the literature if: 1) study types including cohort studies, case reports, case series and revie; 2) acupuncture, psychological supports and other non-pharmaceutical treatment were performed; 3) duplicate publications; 4) non-COVID-19 participants were enrolled.

Information sources: We search the databases including the China National Knowledge Infrastructure (CNKI), PubMed, Wanfang Database, ClinicalTrails.gov, Chinese Clinical Trial Registry (ChiCTR), Embase and International Clinical Trials Registry Platform (ICTRP) for all published RCTs. Search strategies are with terms related to COVID-19, CHM, WM, etc.

Main outcome(s): All efficacy and safety relevant outcomes reported in the included RCTs were checked and summarized. Primary outcomes: total effectiveness rate. Secondary outcomes: the effectiveness relevant index such as symptom improvement, virological outcome, Computerized Tomography (CT) image improvement rate, blood test improvement, and safety relevant index such as total adverse event rate, adverse event rate, worse condition rate and mortality.

Quality assessment / Risk of bias analysis: Two review authors will independently assess the risk of bias for each study using the criteria outlined in the Cochrane Handbook for Systematic Reviews of Interventions . It includes: 1) random sequence generation (checking for possible selection bias); 2) allocation concealment (checking for possible selection bias); 3) blinding of participants and personnel (checking for possible performance bias); 4) blinding of outcome assessment (checking for possible detection bias); 5) incomplete outcome data (checking for possible attrition bias due to the amount, nature and handling of incomplete outcome data) and 6) selective

Li et al. Inplasy protocol 202210072. doi:10.37766/inplasy2022.1.0072 Downloaded from https://inplasy.com/inplasy-2022-1-0072/

reporting (checking for reporting bias); 7) publication status (checking for publication bias). We resolved any disagreement by discussion or by involving a third assessor.

Strategy of data synthesis: Review Manager software (RevMan 5.4.1, 2020) will be used for statistical analysis. For dichotomous data, we presented results as relative risk (RR) ratio with 95% confidence intervals (CIs). For continuous data, we used the mean difference (MD) if outcomes were measured in the same way between trials. We calculated the standardized mean difference (SMD) to combine trials that measure the same outcome, but use different methods. In cases where trial data were missing, we first attempted to contact the original trial investigator to verify the study characteristics and obtain missing information. If the missing data are not available, then we would base on the number randomized minus any participants with missing outcomes. We excluded trials where more than 20% of participants were lost to follow-up. χ2 and I2 quantitative tests were used to test the heterogeneity among the studies. When $P < 0.10$, $I2 > 50\%$, a random-effects model was selected for meta-analysis, and when $P > 0.10$, $I2 < 50\%$, a fixed-effect model was applied. Sensitivity analyses were performed by excluding a study and analyzing the remaining data for each round to test the robustness of our results. Reporting bias (such as publication biases) was reported by using funnel plots in the meta-analysis when the number of trials on an outcome measure was larger than ten.

Subgroup analysis: Subgroup analyses including total effectiveness rate and total adverse event rate of different severity of COVID-19 patients between groups were recorded. We will report the results of subgroup analyses quoting the Chi2 statistic and P-value, and the interaction test I2 value.

Sensitivity analysis: We will carry out sensitivity analysis to explore the effect of trial quality on important outcomes in the review. Where there is a high risk of bias in the allocation of participants to groups associated with a particular study or high levels of missing data, we will explore this by sensitivity analysis. We will use the following outcomes in sensitivity analysis: • Symptom improvement (symptom score of fever, cough, weakness, dry throat & pharyngalgia after treatment) • Virological outcome (time to viralassay conversion) • Blood test improvement(WBC, LYM, LYM%, CRP, RCT after treatment) Sensitivity analyses will be performed by excluding a study and analyzing the remaining data for each round to test the robustness of our results.

Language: English.

Country(ies) involved: China.

Keywords: Systematic review; Meta-analysis; Combined Chinese Herbal Medicine-Western Medicine therapy; Corona Virus Disease 2019 severity.

Contributions of each author:
Author 1 - Lu Li.
Author 2 - Hongliang Xie.
Author 3 - Ling Wang.
Author 4 - Aolin Zhang.
Author 5 - Xuan Mou.
Author 6 - Yifan Lin.
Author 7 - Hongli Ma.
Author 8 - Yu Wang.
Author 9 - Jian Li.
Author 10 - Jingshu Gao.
Author 11 - Chi Chu Wang.
Author 12 - Ping Chung Leung.
Author 13 - Xiaohui Fan.
Author 14 - Xiaoke Wu.

Li et al. Inplasy protocol 202210072. doi:10.37766/inplasy2022.1.0072 Downloaded from https://inplasy.com/inplasy-2022-1-0072/

Keywords: Systematic review; Meta-analysis; Combined Chinese Herbal Medicine-Western Medicine therapy; Corona Virus Disease 2019 severity.

Download PDF - 202210072

Review Status: Completed but not published

Copyright © 2022 INPLASY® Published by INPLASY. All rights reserved
