# Supplementary material for: The efficacy and safety of combined chinese herbal medicine and western medicine therapy for COVID-19: a systematic review and meta-analysis
Source: Chin Med. 2022 Jun 21;17:77. doi: 10.1186/s13020-022-00600-z (PMC9210065; doi:10.1186/s13020-022-00600-z)

**Additional file 3.**

**GRADE assessment for combined CHM-WM vs WM alone treatment**

**Results on Efficacy**

1. Total effectiveness rate


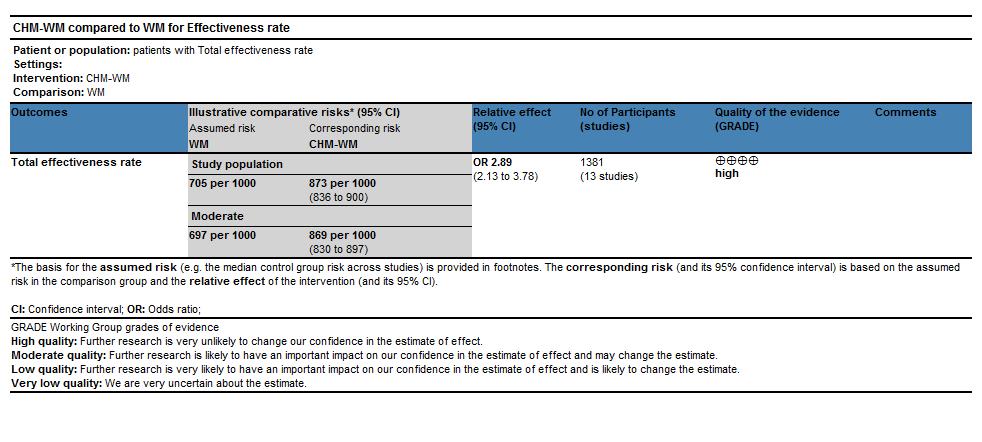


1. Symptom improvement


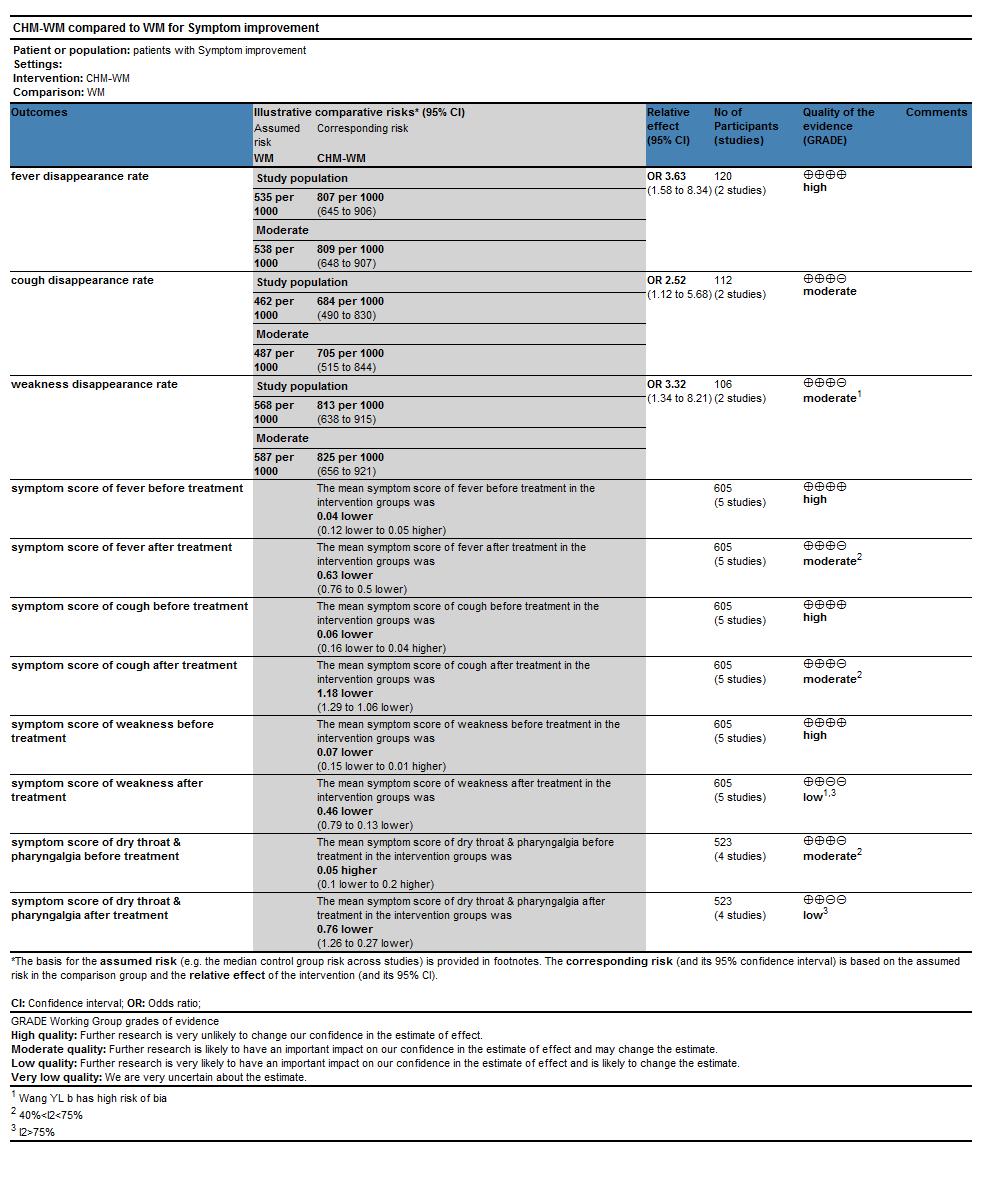


1. Virological outcome


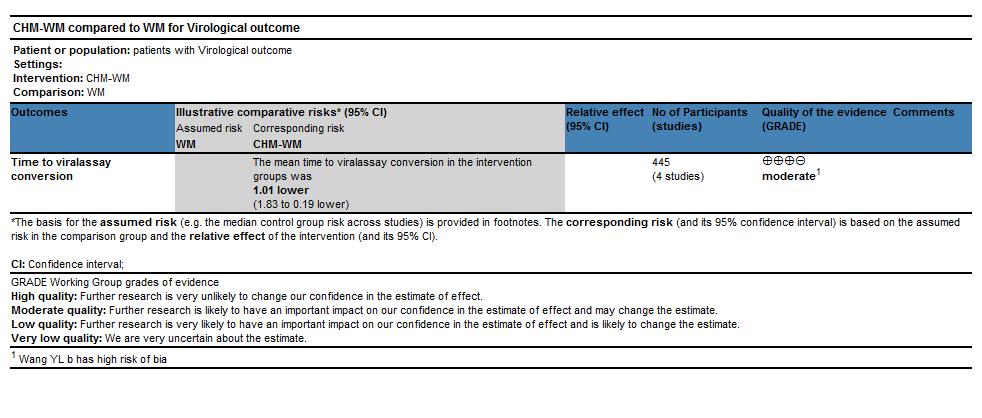


1. CT image improvement rate


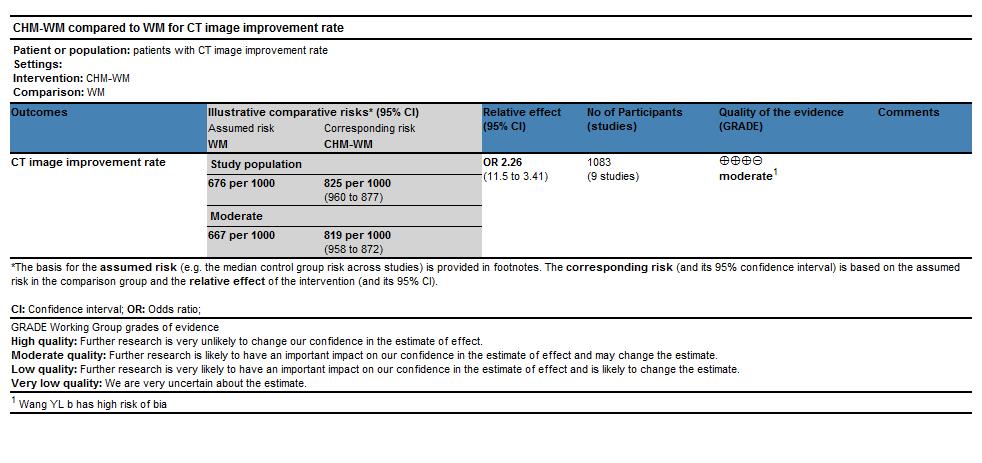


1. Blood test improvement


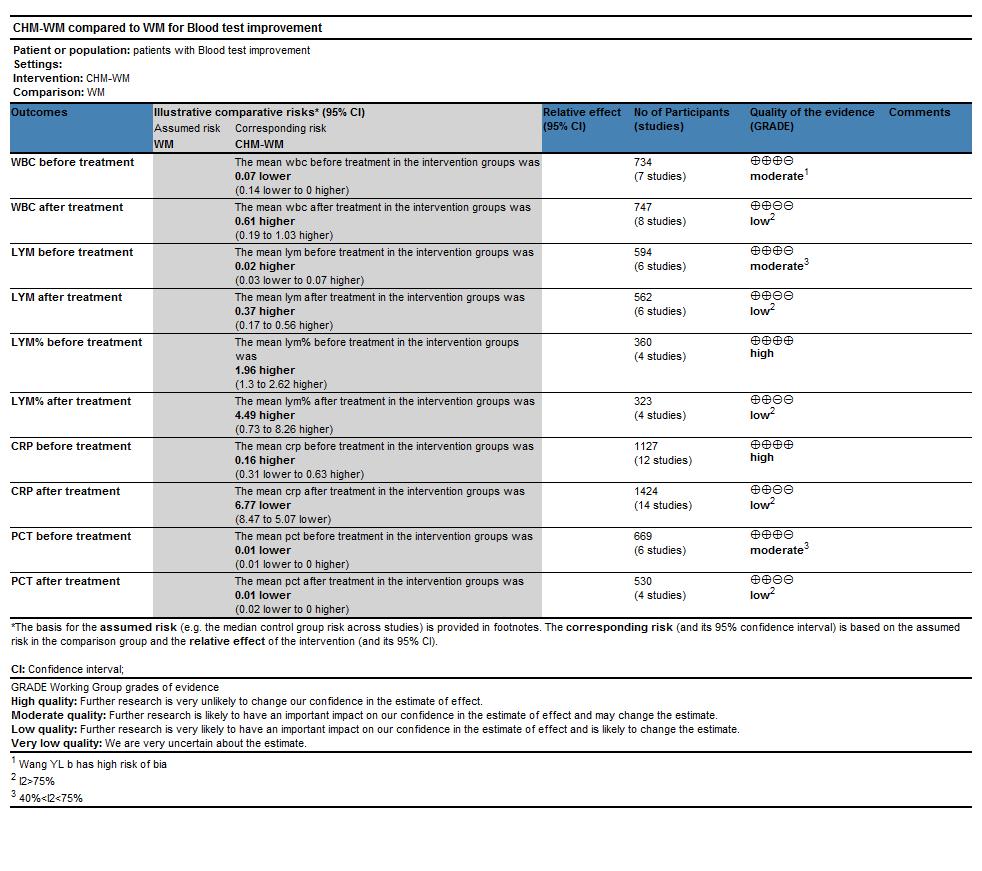


F. Results on Safety


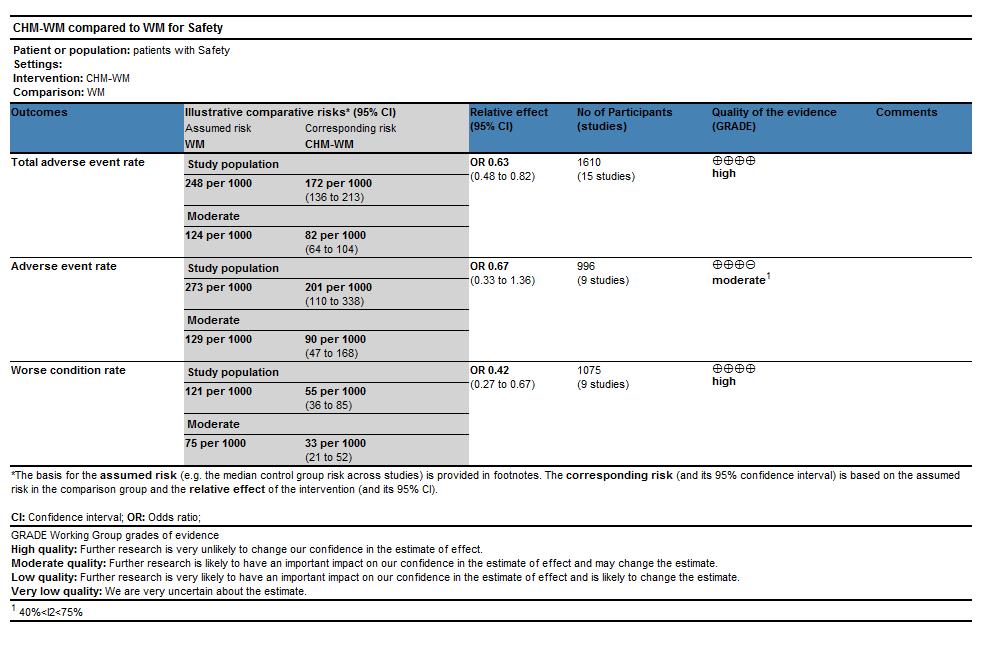

Supplement: Supplementary file 5 — Additional file 5. GRADE assessment for combined CHM-WM vs WM treatment. [file 13020_2022_600_MOESM5_ESM.docx]
